# Evidence That Microorganisms at the Animal-Water Interface Drive Sea Star Wasting Disease

**DOI:** 10.3389/fmicb.2020.610009

**Published:** 2021-01-06

**Authors:** Citlalli A. Aquino, Ryan M. Besemer, Christopher M. DeRito, Jan Kocian, Ian R. Porter, Peter T. Raimondi, Jordan E. Rede, Lauren M. Schiebelhut, Jed P. Sparks, John P. Wares, Ian Hewson

**Affiliations:** ^1^Department of Biology, Estuary and Ocean Science Center, San Francisco State University, Tiburon, CA, United States; ^2^Center for Marine Science, University of North Carolina Wilmington, Wilmington, NC, United States; ^3^Department of Microbiology, Cornell University, Ithaca, NY, United States; ^4^Unaffiliated Researcher, Freeland, WA, United States; ^5^Department of Clinical Sciences, College of Veterinary Medicine, Cornell University, Ithaca, NY, United States; ^6^Institute of Marine Sciences, Department of Ecology and Evolutionary Biology, University of California, Santa Cruz, Santa Cruz, CA, United States; ^7^Life and Environmental Sciences, University of California, Merced, Merced, CA, United States; ^8^Department of Ecology and Evolutionary Biology, Cornell University, Ithaca, NY, United States; ^9^Department of Genetics, University of Georgia, Athens, GA, United States

**Keywords:** sea star wasting, oxygen, heterotroph, remineralization, phytoplankton

## Abstract

Sea star wasting (SSW) disease describes a condition affecting asteroids that resulted in significant Northeastern Pacific population decline following a mass mortality event in 2013. The etiology of SSW is unresolved. We hypothesized that SSW is a sequela of microbial organic matter remineralization near respiratory surfaces, one consequence of which may be limited O_2_ availability at the animal-water interface. Microbial assemblages inhabiting tissues and at the asteroid-water interface bore signatures of copiotroph proliferation before SSW onset, followed by the appearance of putatively facultative and strictly anaerobic taxa at the time of lesion genesis and as animals died. SSW lesions were induced in *Pisaster ochraceus* by enrichment with a variety of organic matter (OM) sources. These results together illustrate that depleted O_2_ conditions at the animal-water interface may be established by heterotrophic microbial activity in response to organic matter loading. SSW was also induced by modestly (∼39%) depleted O_2_ conditions in aquaria, suggesting that small perturbations in dissolved O_2_ may exacerbate the condition. SSW susceptibility between species was significantly and positively correlated with surface rugosity, a key determinant of diffusive boundary layer thickness. Tissues of SSW-affected individuals collected in 2013–2014 bore δ^15^N signatures reflecting anaerobic processes, which suggests that this phenomenon may have affected asteroids during mass mortality at the time. The impacts of enhanced microbial activity and subsequent O_2_ diffusion limitation may be more pronounced under higher temperatures due to lower O_2_ solubility, in more rugose asteroid species due to restricted hydrodynamic flow, and in larger specimens due to their lower surface area to volume ratios which affects diffusive respiratory potential.

## Introduction

Sea star wasting (SSW) disease gained prominence in 2013 when it caused mass mortality of > 20 asteroid species in the Northeastern Pacific (Hewson et al., 2014) with continuous observations since (Miner et al., 2018; Jaffe et al., 2019). SSW in field populations is reported to comprise a wide suite of disease signs, including loss of turgor (deflation), discoloration, puffiness, arm twisting/curling, limb autotomy, body wall lesions and erosions and protrusion of pyloric caeca and gonads (Hewson et al., 2014). There is currently no case definition for any species of asteroid, nor is the progression of disease signs characteristic of SSW (i.e., SSW has no pathognomic signs; Hewson et al., 2019). Lesions compatible with SSW in various asteroid species have been reported since at least 1896 in the Eastern United States (Mead, 1898), and at several locations globally (reviewed in Hewson et al., 2019). SSW affects larger asteroids more than smaller asteroids (Hewson et al., 2014; Eisenlord et al., 2016), and resulted in shifts in size structure after SSW mass mortality from larger to smaller individuals of *Pisaster ochraceus*, which was believed to be due to recruitment of juveniles (Bates et al., 2009; Eisenlord et al., 2016; Menge et al., 2016; Kay et al., 2019).

The cause of SSW is unresolved. Early reports that SSW was associated with a densovirus (Hewson et al., 2014) were refuted by subsequent investigation that failed to show a consistent association between the virus and presence of disease (Hewson et al., 2018, 2020), and recent description of persistent and phylogenetically widespread infection by related densoviruses (Jackson et al., 2020a,b) suggest this virus to be a component of normal microbiome. Furthermore, SSW is not consistently associated with any bacterial or microbial eukaryotic organism (Hewson et al., 2018). Environmental conditions, including elevated water temperatures (Eisenlord et al., 2016; Kohl et al., 2016), lower water temperatures and higher pCO_2_ (Menge et al., 2016), and meteorological conditions (Hewson et al., 2018) correspond with SSW at distinct locations. Recent modeling studies suggest repeated sea surface temperature anomalies may correlate with SSW (Aalto et al., 2020). Reports of SSW spread between adjacent geographic locations, through public aquarium intakes, and challenge experiments with tissue homogenates suggested a transmissible etiology (Hewson et al., 2014; Bucci et al., 2017). However, there is a lack of mechanistic understanding how SSW is generated in affected individuals.

**FIGURE 1:**
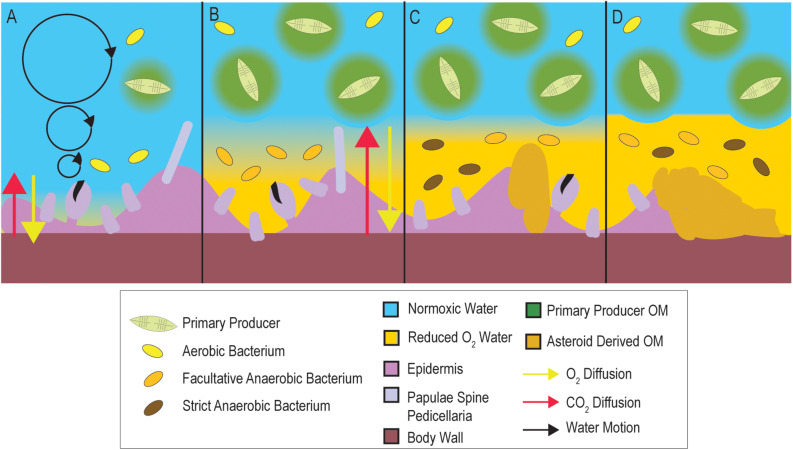
Conceptualization of how microbial activities in the DBL may precipitate SSW. Under typical conditions **(A)**, DBL conditions are normoxic. When OM increases in overlying waters (**B**; e.g., during elevated primary productivity, from terrestrial runoff, or from decaying asteroid carcasses), microbial heterotrophic respiration and cell abundance are stimulated, which may result in the formation of depleted O_2_ within the DBL. This in turn results in longer distances over which diffusion must occur to maintain animal respiratory demand. Over time **(C)** depleted O_2_ conditions in the DBL results in tissue damage, and prevalence of strict and facultative anaerobes. Because their growth is less efficient than aerobic metabolisms their abundance is less than at onset of depleted O_2_ conditions. Release of labile OM from decaying tissues **(D)** and persistent OM-rich conditions within the asteroid DBL result in animal mortality.

All aquatic surfaces are coated with a thin film of water (i.e., diffusive boundary layer; DBL) that impedes gas and solute exchange, and, provided aerobic respiration is sufficiently high, depleted O_2_ conditions can form on a surface despite O_2_ saturated water circulating above (Jørgensen and Revsbech, 1985). We propose that SSW is a sequela (i.e., condition that is the consequence of previous insult) of microbial activities within the DBL which may result in O_2_ diffusion limitation (Figure 1). Elevated organic matter (OM) concentrations may stimulate the growth of copiotrophic microorganisms in the BDL, driving down dissolved O_2_ concentrations and causing depleted O_2_ (i.e., less than saturated) conditions. Over time, these conditions may not meet respiratory O_2_ demand of tissues, resulting in their damage and decomposition, which further enriches near-asteroid pools of OM. This in turn may result in the proliferation of anaerobic taxa on and within tissues. We studied microbiome composition during OM amendment and as animals waste in the absence of external stimuli. Further, we tested whether SSW can result from depleted O_2_ water column conditions. Next, we examined if SSW susceptibility was related to inherent asteroid properties, including rugosity (a key physical characteristic influencing DBL extent) and animal size. We examined whether species with greater respiratory O_2_ demand relative to their calculated O_2_ flux would be more susceptible to SSW. We explored the relationship between primary production, physico-chemical conditions, and SSW during a 5 year time series at a field site. Finally, we investigated stable isotopic signatures of SSW-affected asteroids from the 2013 to 2014 mass mortality event to gain insight into microbial metabolism occurring in concert with mass mortality at the time. The results of these studies collectively suggest that SSW is driven by microorganisms occurring within the DBL and that OM supply may influence SSW development.

## Materials and Methods

Because many sea star wasting (SSW) disease signs are subject to observer bias, we standardized occurrence of SSW between experiments and surveys as the appearance of non-focal body wall lesions across all experiments. The time between experiment initiation and body wall lesion appearance (i.e., lesion genesis) was used as a standardized parameter to assess the speed of SSW. In all experiments, lesion position was standardized by clockwise naming of rays from the madreporite. Individual asteroids were considered dead when tube feet did not move during observation for 30 s, or when all limbs autotomized away from the central disc. The mass of individuals was determined using a top pan balance, and each ray measured from tip to the center of central disc using a ruler or calipers.

### Testing the Impacts of OM Enrichment on *Pisaster ochraceus*

We tested whether OM enrichment caused shifts in bacterial abundance and composition. Twenty *P. ochraceus* (mass 303 ± 26 g) were obtained from the jetty at Bodega Bay in July 2019 and transported to the Bodega Bay Marine Lab (UC Davis), where they were placed in flow-through large volume sea tables for 7 days prior to commencement of the experiment. Asteroids were placed individually into baskets (holes 4 mm diameter at a density of ∼2 holes cm^–2^) and suspended in each of 4 sea tables with flow rates of 60 ± 15 mL s^–1^. A dense (∼10^4^ cells mL^–1^) culture of *Dunaliella tertiolecta* was prepared in 20 L artificial seawater, which was filtered onto 0.2 μm Durapore filters and resuspended in 1 L artificial seawater. The resuspended matter was divided into 21 × 45 mL aliquots and frozen prior to use. Coastal particulate OM (POM) was prepared by filtering 40 L seawater from the unfiltered intake pipe at Bodega Marine Laboratory through 0.2 μm Durapore filters, which was then resuspended in 1 L seawater. This was also divided into 21 × 35 ml aliquots and frozen before use. Individuals (*n* = 5 per treatment in separate sea tables) were amended with 3 OM sources: (1) peptone (˜300 mg individual^–1^ daily); (2) 45mL of *Dunaliella tertiolecta* filtered culture daily; and (3) Coastal POM (6 mL) daily. Individual asteroids were observed daily (aboral and ventral surface) for the presence of lesions and they were weighed. Daily samples (2 mL of water) for surface bacterial abundance were withdrawn using 3 mL syringes which were pressed onto the aboral surface of individual specimens while immersed, and preserved in 5% formalin and kept at 4°C in darkness prior to processing. Surface microbial communities were sampled every 48 h by collecting a swab (sterile cotton-tipped, dry transport system; Puritan) from each aboral surface which were then frozen at −20°C prior to processing. Microbiome compositional analyses was performed as described later in this section.

### SSW Progression in the Absence of External Stimuli

To test whether SSW lesions were preceded or accompanied by shifts in bacterial + archaeal assemblages inhabiting tissues (i.e., microbiomes), we performed two longitudinal studies examining epidermal (here defined as microorganisms inhabiting tissue surfaces as well as those beneath the epidermis and cuticle but excluding body wall and mutable collagenous tissue-associated microorganisms) and body wall (which includes epidermal as well as body wall and mutable collagenous tissue) tissues of specimens that wasted in the absence of external stimuli (i.e., without experimental perturbation and minimal initial sampling).

Epidermal microbiomes were examined in *P. ochraceus* specimens from central California collected on 19 July 2018 (Hewson et al., 2020). Six specimens (mean mass 290 ± 54 g and ray length 11.2 ± 0.9 cm) were collected from the intertidal zone at Davenport, CA (37°1′19″, 122°12′56″W) at low tide and transported in insulated coolers to the Long Marine Laboratory at UC Santa Cruz, where they were housed in flow-through aquaria (3.81 ± 0.05 mL s^–1^; mean water residence time 37 min) indoors in individual containers. Individuals were maintained under these conditions for the duration of the experiment. Temperature and salinity were measured daily using a YSI-3000 handheld meter. After a 48 h acclimation period, a small scar (∼5 mm long) was made on a single ray using a sterile 4 mm biopsy punch. Individual asteroids were monitored daily for the presence of lesions (as defined above). A small tissue sample (∼3 mm × 2 mm) was taken from the margin of artificial scars using sterile 4 mm biopsy punches after 24 h. New artificial scars were made on adjacent rays each day, and sampled after 24 h. When lesions away from these artificial scars were observed (i.e., lesions not caused by physical scarring), their margin tissues were sampled using the same approach. All tissue samples were placed into sterile 1.2 mL cryovials and immediately frozen in liquid N_2_ or in a −80°C freezer.

To examine body wall microbiome shifts (i.e., biopsy punch), *P. ochraceus* specimens were collected on 24 May 2018 from Davenport, CA (37°1′19 ″N, 122°12′56″W). The specimens were transported in an insulated container to the Long Marine Laboratory at UC Santa Cruz where they were placed into a single, flow-through sea table. The individuals were measured, weighed and photographed to fingerprint apical circlet pattern (individual specimens have distinctive pattern of spines/paxillae on the aboral surface of the central disc, which facilitates identification). A single biopsy punch (4 mm) was retrieved from each individual and preserved in liquid N_2_. Upon appearance of lesions and at time of death, lesions were sampled by biopsy punch and preserved in liquid N_2_.

### Testing the Impact of Depleted O_2_ Conditions on SSW in *Asterias forbesi*

To test whether SSW could result from depleted O_2_ conditions, we incubated asteroids in incubations where O_2_ was depleted and examined the abundance and composition of bacteria + archaea that occurred on their surfaces. Twenty-four *Asterias forbesi* (mass 63 ± 7 g) were obtained from Bar Harbor, ME (44°25.7′N, 68°12.0′W) on 29 June 2019 and transported in insulated coolers to the laboratory at Cornell University. There, the individuals were initially placed into a single, 320 L aquarium for 24 h, before being placed into individual flow-through baskets and divided into two treatments. One large volume (230 L) sump containing artificial seawater (Instant Ocean) was set up for each treatment. One sump served as a control, while another sump was continuously sparged with medical-grade N_2_ (∼5–6 L min^–1^) to deplete O_2_. Continuous flow between sump and aquarium systems was maintained by non-self-priming pumps and removed by gravity through a standpipe. O_2_ and temperature was continuously monitored in the experimental tanks using HOBO O_2_ Loggers (U26; Onset). Individual asteroids were monitored daily for the gross appearance of lesions on aboral and ventral surfaces. At experiment initiation, surface bacterial abundance samples were taken and preserved as described above. The mass of individuals was recorded daily; specimens remained without prey during the experiment. Surface microbial swabs were sampled every 48 h following the approach outlined above. To reduce sampling stress on individuals, half of individuals in each treatment were biopsied (2 × 3 mm biopsy samples collected using sterile biopsy punches) on their aboral surface at experiment initiation, and then every 120 h until experiment termination (biopsied specimen lesion genesis time was not significantly different to non-biopsied specimen lesion genesis time). Biopsy punches (1 each) were preserved in RNALater or in 10% neutral buffered formalin. Upon appearance of lesions, their margins were sampled using a 5 mm biopsy punch to scrape a ∼ 3 mm × 2 mm tissue sample. Additionally, a 3 mm biopsy punch was used to obtain a sample through body wall tissues on the lesion margins. Subsequent analysis of biopsy punch samples was not performed as part of this study, and all microbial composition data was derived from surface swabs. Lesion margin tissues were stored at −20°C until analysis.

### Determination of Bacterial Cell Abundance

Bacterial abundance in depleted O_2_ and OM amendment experiments was determined by SYBR Gold staining and epifluorescence microscopy (Porter and Feig, 1980; Noble and Fuhrman, 1998; Shibata et al., 2006). An aliquot (1 mL) of each sample was first stained with SYBR Gold (2 μl mL^–1^ of the 10,000X stock) for 2 min, then samples were filtered through 25 mm diameter 0.2 μm black cyclopore filters mounted on 25 mm Type AA Millipore filters to even flow. The filters were removed from the backing filter, adhered to clean glass slides and mounted in 30 μL of PBS:Glycerol (50:50) containing 0.1% p-phenylenediamine. The slides were visualized on an Olympus BX-51 epifluorescence microscope under blue light excitation. Over 200 cells were counted in > 10 fields. Bacterial abundance was calculated by multiplying mean abundance per reticle grid by total grids per filter area, and divided by volume passed through the filter. For some samples, high background fluorescence precluded accurate counts and so are not included in downstream analyses.

### Microbial Assemblage Analyses and Bioinformatic Treatment

Microbial assemblages inhabiting body wall samples (i.e., biopsy punch; SSW progression in the absence of external stimuli), lesion margins (i.e., epidermal scrapes; SSW progression in the absence of external stimuli), and at the animal/water interface (i.e., swabs; depleted O_2_, and OM enrichment experiments) were examined by 16S rRNA amplicon sequencing. Nucleic acids were extracted from frozen 3 mm biopsies, lesion margin scrapes, and frozen surface swabs using a Quick-DNA Fungal/Bacterial MiniPrep Kit (Zymo Research, cat# D6005) according to the manufacturer’s protocol. Bacterial DNA was quantified using a Quant-IT dsDNA Assay (Invitrogen, cat# Q33120) in conjunction with a StepOnePlus^TM^ Real-Time PCR system (Applied Biosystems). Bacterial community composition was examined via PCR amplification sequencing of the V4 region of the 16S rRNA gene using a modified version from Caporaso et al. (2011). Fifty microliter PCR reactions contained 1 × 5PRIME HotMasterMix (QuantaBio, cat# 2200400) and 0.1 μM each primer (515f/barcoded-806r). Template DNA quantity varied between experiments. For examining microbiome composition during SSW progression in the absence of external stimuli, 1 μl of extract [containing from below detection limit (0.1 ng) to 80 ng (mean = 17 ng)] was used as PCR template. For *Asterias forbesi* hypoxia and *Pisaster ochraceus* OM enrichment, 5 pg of genomic DNA (determined by Femto Bacterial DNA Quantification Kit; Zymo Research, cat# E2006) was used to standardize prokaryotic template amounts (Hewson, 2019). PCR products from duplicate reactions were pooled for each sample and cleaned using a Mag-Bind RxnPure Plus Kit (Omega Bio-tek, cat# M1386-01). Ten ng of bacterial DNA from each sample were pooled, libraries prepared using the NextFLEX prep, and sequenced on 2 lanes of Illumina MiSeq (2 × 250 bp paired end) at the Cornell Biotechnology Resource Center. Sequence libraries are available at QIITA under studies 12131 and 13061 and at NCBI under BioProject PRJNA637333.

Raw sequences were uploaded to QIITA and processed using the native 16S rRNA pipeline (Gonzalez et al., 2018). After demultiplexing, reads were trimmed to 150 bp and sub-OTUs (sOTUs) were configured using Deblur (Amir et al., 2017). The SEPP phylogenetic tree and BIOM/FA files were downloaded from the deblur reference hit table and converted into qiime2 (v2019.10) artifacts, after which taxonomy was assigned using the Silva 132 release (Quast et al., 2012) and q2-feature-classifier plugin. All files were then imported into R (v3.6.1) using qiime2R v(0.99) (Caporaso et al., 2010) and compiled into a phyloseq (v1.28) (McMurdie and Holmes, 2013) object for downstream analyses.

Amplicon data was transformed using the PhILR (Phylogenetic Isometric Log-Ratio Transform, v1.1) package for ordination (Silverman et al., 2017). PhILR transforms compositional data (i.e., proportional, or relative data) into a new matrix of “balances” that incorporates phylogenetic information. Here, a balance is defined as the isometric log-ratio (ILR) between two clades that share a common node. For each experiment, low abundance sOTUs were filtered based on sequencing depth/evenness/rarefraction and a pseudocount of 0.65 was applied to all 0 counts. The ILR analyses in this study do not require an even sampling depth, and therefore, count normalization techniques like rarefying, which lead to a loss of information (McMurdie and Holmes, 2014), were not utilized. Phyloseq (1.32.0) (McMurdie and Holmes, 2013) was used to perform principal coordinate analyses (PCoA) on the Euclidean distances between PhILR transformed samples, and the adonis function in the vegan package (v2.5-6) (Oksanen et al., 2019) was used to perform a PERMANOVA on relevant PCoAs. For June 2018 *Pisaster ochraceus* samples, a principal coordinate analysis based on Weighted Unifrac distances (Lozupone et al., 2011) was used *en lieu* of the PhILR transformation due to higher explained variance from a PERMANOVA. For these samples, low abundance sOTUs were removed (< 5) based on a rarefaction curve and the data was converted to relative abundance. Multiple filtering strategies were applied to all analyses and did not affect results.

Balances were used to quantify differential abundance in order to address the issue of compositionality in amplicon data. Two approaches were used that rely on the ILR transformation. For comparisons between two categorical variables (diseased/non-diseased tissue and surface swabs from specimens immediately before lesions appear with earlier specimens), a sparse logistic regression with an *l*_1_ penalty of λ = 0.15 was applied to PhILR balances using the glmnet package v(3.0-2) (Friedman et al., 2010). For time-course experiments, the PhyloFactor package (v0.0.1) was used. PhyloFactor calculates balances in a similar fashion to PhILR, but instead of using nodes to contrast clades, Phylofactor bisects a phylogenetic tree along its edges in an iterative manner. Each iteration, or “factor,” was regressed using a generalized linear model. Edges were maximized using the F statistic and a Kolmogorov-Smirnov test was used to break the iterations. The O_2_ physiology of bacterial taxonomic units observed in this study were assigned based on descriptions in Bergey and Holt (2000), and copiotrophic assignment based on descriptions in Haggerty and Dinsdale (2016).

### Additional Experimental Challenges of *Pisaster ochraceus*

We tested whether SSW could result from variable water flow rates, desiccation, and challenge with asteroid tissue-derived material. *P. ochraceus* (*n* = 24) were collected from Mitchell Cove, Santa Cruz (36°57.1N, 122°2.51′W), on 25 June 2018 at low tide (mean mass 357 ± 37 g), and transported in a cooler to the Long Marine Laboratory. Metadata on their size and weight, along with mean flow rates in incubations and change in mass over time is provided in Supplementary Table S1. The temperature in aquarium settings for all experiments was measured by Onset Hobo Spot loggers (*n* = 2) which were deployed into aquarium outflows for the first 96 h of the experiment, and after 144 h was measured using a YSI Handheld Instrument.

The impact of water flow on SSW was examined in 12 specimens. Asteroids (*n* = 6 each treatment) were placed into individual plastic boxes which were subject to high (7.06 ± 0.39 ml s^–1^; water residence time in container ∼ 20 min) and low (2.84 ± 0.26 ml s^–1^; water residence time 50 min) flow-through rates. Temperature and salinity were monitored daily using a handheld YSI Probe (YSI-3000). Individuals were visually inspected every 24 h for the presence of body wall lesions and were weighed to determine changes in their overall mass over the course of the experiment.

The impacts of desiccation under both high and low flow was examined by first placing 6 individuals onto plastic trays in sunlight for 1 h. During this period, air temperature was 33.8°C (mean flow-through incubation temperature was 14.5°C). After desiccation for 1 h, individuals were placed into individual flow-through plastic boxes and monitored per the variable flow rate experiments described above.

The effects of challenge with SSW-affected tissue homogenates was examined in 4 specimens. A single SSW-affected star was collected at Davenport, CA (37°1′19″N, 122°12′56″W) at low tide on 25 June 2018 and transported to the Long Marine Laboratory. Tissue surrounding lesions (∼2 g total) was excised using a sterile razor blade, and placed into 40 mL of seawater from the lab’s inflow system. The tissue was then homogenized in a sterilized mortar and pestle for 10 min. Half of the tissue homogenate was treated with 10,000U of proteinase k (Sigma-Aldrich) and incubated for 1 h at 37°C to digest proteins in tissue homogenates and permit comparison between protein-bearing and protein-depleted materials. Two *Pisaster ochraceus* were inoculated with 10 mL of crude tissue homogenate and two *P. ochraceus* inoculated with the proteinase-k treated homogenate by direct injection into their coelomic cavity. The inoculated asteroids were then placed in individual plastic flow-through aquaria (under low-flow conditions). Individuals were monitored per the flow-rate experiments described above. Comparison between desiccation and tissue homogenate challenge (described below) were performed against controls high/low flow as described above.

### Modeling of SSW Speed (Lesion Genesis) With Experimental Parameters

The time to SSW development (i.e., lesion genesis time) was modeled across all experiments against available parameters, which varied between experiments (Supplementary Table S2). All statistical analyses were performed using XLStat version 2019.4.1 in Microsoft Excel. Response of lesion time to treatment in OM addition experiments were examined by least squares mean ANOVA. The relationship between lesion time and comparison variables was performed by multiple linear regression, forward or backward selection procedure and change in Akaike’s AIC as entry criterion.

### Comparison of Asteroid Volume and Rugosity With SSW Susceptibility

To test the hypothesis that SSW susceptibility was related to species rugostity (i.e., corrugated-ness, which is a key determinant of boundary layer extent), individual, intact whole-animal specimens of asteroids were collected from several locations (Supplementary Table S3) and immediately preserved in 20% neutral buffered formalin. All individuals were transported to the lab at Cornell University. Computed tomography was initially performed on whole specimens at the Cornell University Equine Hospital without contrast to estimate surface area: volume using a Toshiba Aquillon computed tomographic multi-slice scanner. The relative rugosity between SSW-affected and less SSW-affected asteroid species was examined by calculating the ratio of 3D (determined by computed tomography) to 2D (as calculated below) for each asteroid specimen and comparing between species. Asteroid species were categorized based on prevalence of SSW (less or not affected = *Dermasterias imbricata*, *Henricia leviuscula*, *Patiria miniata*; SSW affected = *Pisaster ochraceus*, *Solaster stimpsoni*, *Pycnopodia helianthoides*, *Leptasterias* sp., *Asterias forbesi*, *Orthasterias kohleri*, *Pisaster giganteus*, and *Pisaster brevispinus*) as reported elsewhere (Montecino-Latorre et al., 2016; Bucci et al., 2017; Miner et al., 2018; Jaffe et al., 2019; Konar et al., 2019). These were likewise compared between animal volume, surface area:volume and 2D area.

Because the resolution of computed tomography is only 400 μm, which is larger than some surface features (e.g., papulae), we performed micro-CT (μCT) analyses on one large and one small individual of several key species after staining for at least 24 h in IKI solution (Supplementary Figure S1). X-Ray μCT data were analyzed using the Avizo version 2019.4 software (Thermo Fisher Scientific). Briefly, 2-D image slices were uploaded and stacked to reconstruct a 3-D volume for each specimen. A median filter was applied to each 3-D reconstruction to reduce noise and smooth edges. The volume of interest was isolated from surrounding background using a thresholding approach. Next, the total volume for each specimen was segmented into 1 cm sections (each composed of 500 stacked 20 μm slices). After eliminating holes from each 1 cm sub-volume, the total volume, surface area, and rugosity were determined using the “Label Analysis” module of the Avizo software. The surface areas reported were calculated by subtracting the 2-D surface areas of the two, flat end slices from the total 3-D surface area for each 1-cm segment. The relationship between ray length and surface area was investigated by linear regression. We first calculated total two-dimensional area for each specimen by taking into consideration central disc radius and assuming triangular shape of rays, accounting for total height of central disc and height of ray tip (see Supplementary Figure S2). We then used the ratio of total surface area (determined by CT) to calculate surface area as a measure of rugosity. All image stacks for micro-CT analysis are available from MorphoSource (Duke University) under sample identifiers S32007, S32014, S32018, S32021, S32022, S32029, S32031, S32036, S32037, S32039, S32042, S32046, S32047, S32048 and S32050.

### Asteroid Respiration

The respiration rates of individual asteroids was measured upon experiment initiation for *Asterias forbesi* and *Pisaster ochraceus*, and for additional species at the Bodega Marine Laboratory. HOBO O_2_ Loggers (U26; Onset) were placed into sealable plastic containers to which individuals were added. The incubations were circulated using a battery-operated submersible DC motor and propeller. The containers were then filled by immersion in flow-through seawater, and sealed, excluding all visible bubbles. Individuals were incubated for 1–2 h in containers before retrieval of O_2_ probe. Respiration rate was calculated by the linear change in O_2_ concentration over time in incubations. Respiration rates were compared to calculated maximum diffusion rates based on overall surface area determined by computed tomography using Fick’s second law of diffusion (J = −D^∗^∂C/∂d where J = flux across the membrane, D = diffusivity constant of O_2_ in seawater, C = concentration difference between coelomic fluid and seawater—in this case assuming completely anoxic coelomic fluid and saturated seawater, and d = thickness of outer epithelium -assumed to be 20 μm.).

### Time Series Analyses of SSW Intensity and Chlorophyll a at Whidbey Island

To understand the relationship between primary producer biomass (chlorophyll a), physico-chemical parameters (temperature, salinity, dissolved O_2_), and occurrence of SSW, we examined data obtained from the Penn Cove Shellfish data buoy (48.2191N, 122.7048W; accessed through the NVS Data Explorer^1^) and compared this to observations of SSW frequency at Coupeville Wharf as reported previously (Hewson et al., 2018) from August 2014 to June 2019 (i.e., 5 years). The data buoy and dive site lie at Coupeville are ˜600 m apart. Diver surveys reported in Hewson et al. (2018) were performed in water depths < 8 m. We also compared SSW frequency with precipitation data obtained from the National Center for Environmental Information (NOAA), which may be seen as a proxy for potential terrestrial runoff. We first calculated the mean time of SSW over the 5 year period and compared this to 5 year mean values of all parameters. We then performed at-time-of-SSW to prior to SSW comparison following a shifting window approach comparing the 3 month window immediately before SSW with 3 month windows in earlier months.

### Stable Isotopic Signatures in Historical SSW-Affected Asteroid Specimens

The natural abundance of ^15^N and ^13^C was determined in 71 individual starfish specimens, including 50 individuals representing paired grossly normal/SSW-affected species at distinct sites and sampling times which were collected as part of prior work (Hewson et al., 2014; Supplementary Table S4). We included an additional 21 individuals of different species to provide context of stable isotopic composition. Samples were collected and frozen at −20°C prior to analysis. Thawed tissue samples were subsectioned for analysis by scraping tube feet into sterile 1.2 mL cryovials. Samples were freeze-dried at −45°C for 1 week then ground with mortar and pestle. A subsample of 1 mg of tissue was encapsulated into tin and subsequently analyzed on a Carlo Erba NC2500 Elemental Analyzer coupled to a Thermo Scientific Delta V Advantage IRMS (Bremen, Germany).

## Results

### Impact of OM Enrichment on SSW in *Pisaster ochraceus*

**FIGURE 2:**
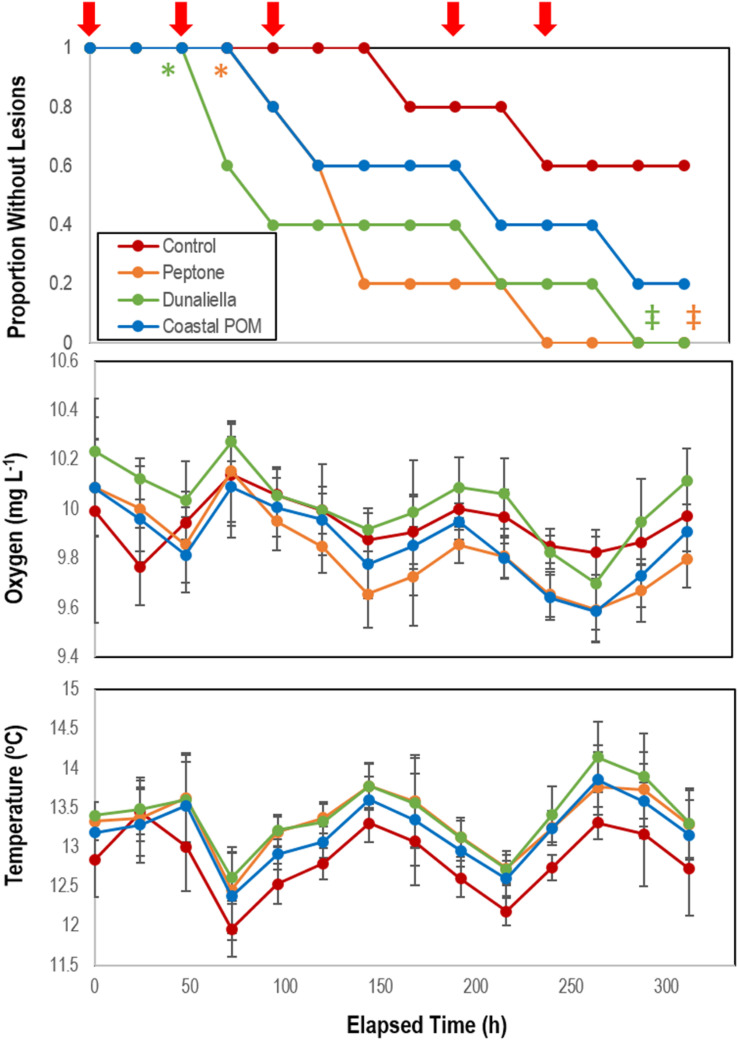
Proportion of grossly normal *P. ochraceus* (*n* = 5 each treatment) incubated in flow-through conditions at the Bodega Marine Lab in response to organic matter enrichments (Peptone, *Dunaliella tertiolecta* culture POM, and coastal POM collected from the inflow at the Bodega Marine Laboratory in August 2019). Dissolved O_2_ and temperature were measured in flow-through sea tables bearing each OM treatment. Error Bars = SE. The red arrows above the top panel indicate sampling for microbiome analyses. * indicates wasting speed (i.e., time to appearance of first lesion) was significantly (*p* < 0.05, Student’s *t*-test) faster than control. ^‡^indicates that the overall trend in lesion formation was significantly different to controls (*p* < 0.05, log-rank test).

The addition of organic substrates (peptone and *Dunaliella tertiolecta*-derived POM) induced significantly faster lesion genesis than control incubations (*p* = 0.012 for peptone and *p* = 0.04 for *Dunaliella*-POM, Student’s *t*-test, *df* = 5), but lesion genesis time was not significantly different for the addition of coastal-POM (Figure 2). Collective treatment temporal pattern of lesion genesis was only significantly different from controls with amendment with peptone (*p* = 0.0154, log-rank test, *df* = 5) and *Dunaliella*-POM (*p* = 0.0339, log-rank test, *df* = 5). Dissolved O_2_ in incubations varied over the course of the experiment from 9.6 to 10.2 mg L^–1^ and were never under-saturated. Temperature varied from 12 to 14°C, but variation did not correspond with SSW in any treatment. There was no significant difference in the rate of animal mass change over the course of the experiment between treatments (Supplementary Figure S3), where individuals lost 11.0 ± 0.2% of their initial mass over the course of the experiment.

**FIGURE 3:**
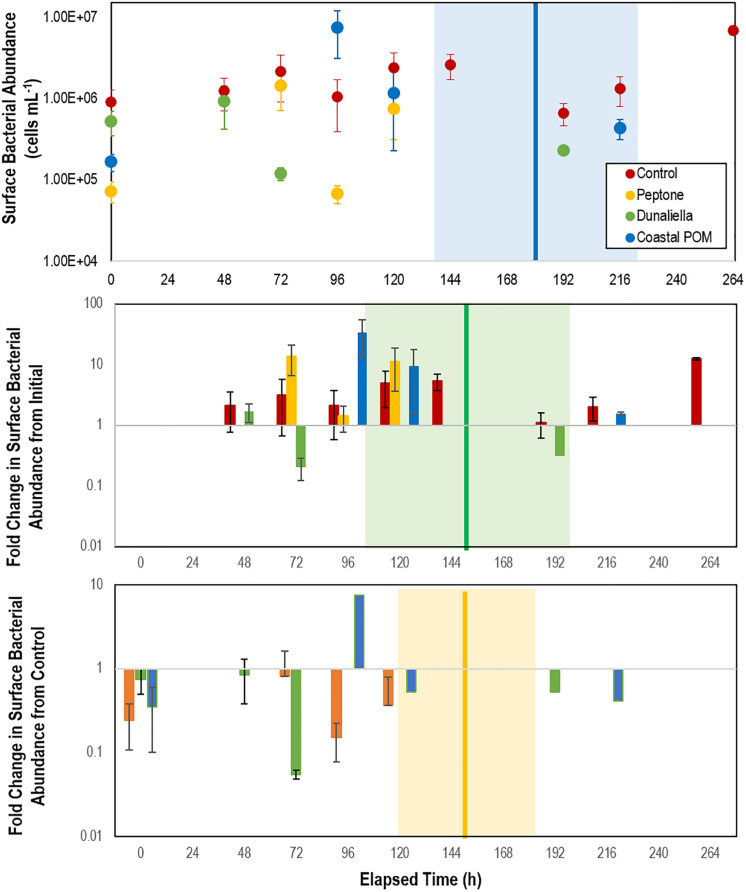
Abundance of bacteria in proximity to *P. ochraceus* surfaces (top), fold change from initial (middle), and relative to controls (bottom). During first 10 days of experiment in response to organic matter enrichment (*n* = 5 for each treatment) as assessed by SYBR Gold staining and epifluorescence microscopy. Control specimens are indicated in red, while the mean of specimens that wasted in Peptone, *Dunaliella tertiolecta* POM, and Coastal POM are indicated separately. The solid vertical line on the top panel represents the mean time that asteroids developed lesions in the coastal POM treatment, the solid line on the middle panel represents the mean time for lesion development in *Dunaliella tertiolecta* POM treatments, and solid line on the bottom panel represents the mean time for lesion development in peptone treatments (separated between panels for clarity). The shaded regions represent lesion development standard error for respective treatments.

Bacterial cell abundance on surfaces (relative to both initial values and controls) illustrated large swings prior to SSW onset (Figure 3). Individuals that did not waste over the course of the experiment maintained abundances of 0.7–2.6 × 10^6^ cells mL^–1^, which were enriched 53–1,743% above aquarium water bacterioplankton abundances in incubation treatments (Figure 3). In aggregate, SSW-affected specimens had higher bacterial abundances than grossly normal specimens (Supplementary Figure S4), however, the relationship was not significant because of high variation between treatments with OM. Treatment bacterial abundances remained no different from controls over the first 48 h of incubation, but increased relative to controls in peptone and coastal-POM treated asteroids after 72 and 96 h, respectively. However, by 96 h for peptone and 120 h for coastal POM both amendments had again declined, and remained no different from controls after this time (Figure 3). In contrast, bacterial abundance in *Dunaliella*-POM incubations were no different to controls over the first 48 h of incubation, and were far less than controls after 72 h.

**FIGURE 4:**
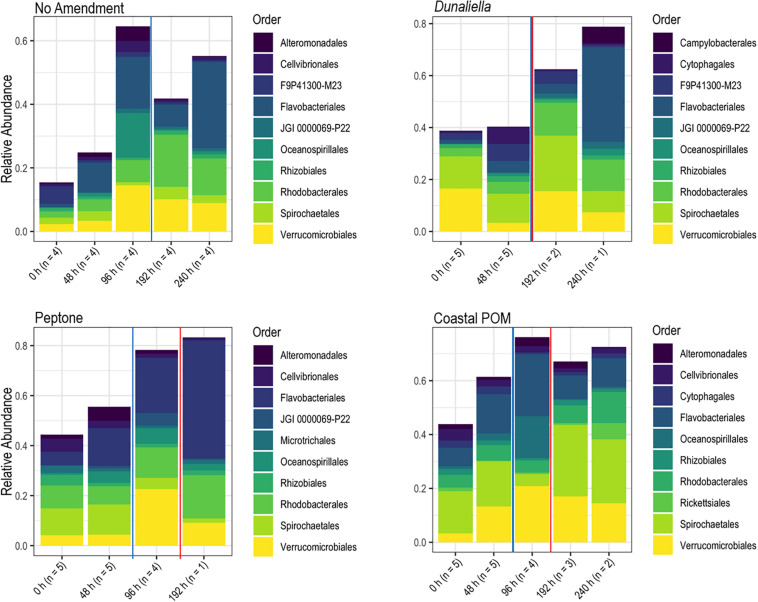
Relative abundance of bacterial orders derived from *P. ochraceus* epidermal swabs. Specimens were enriched with the indicated organic material and sampled until lesion genesis. n values reflect the number of healthy specimens at each given timepoint. The solid blue line on each panel indicates when lesions first formed per treatment, and the solid red line on each panel indicates the mean lesion time within the treatment.

**FIGURE 5:**
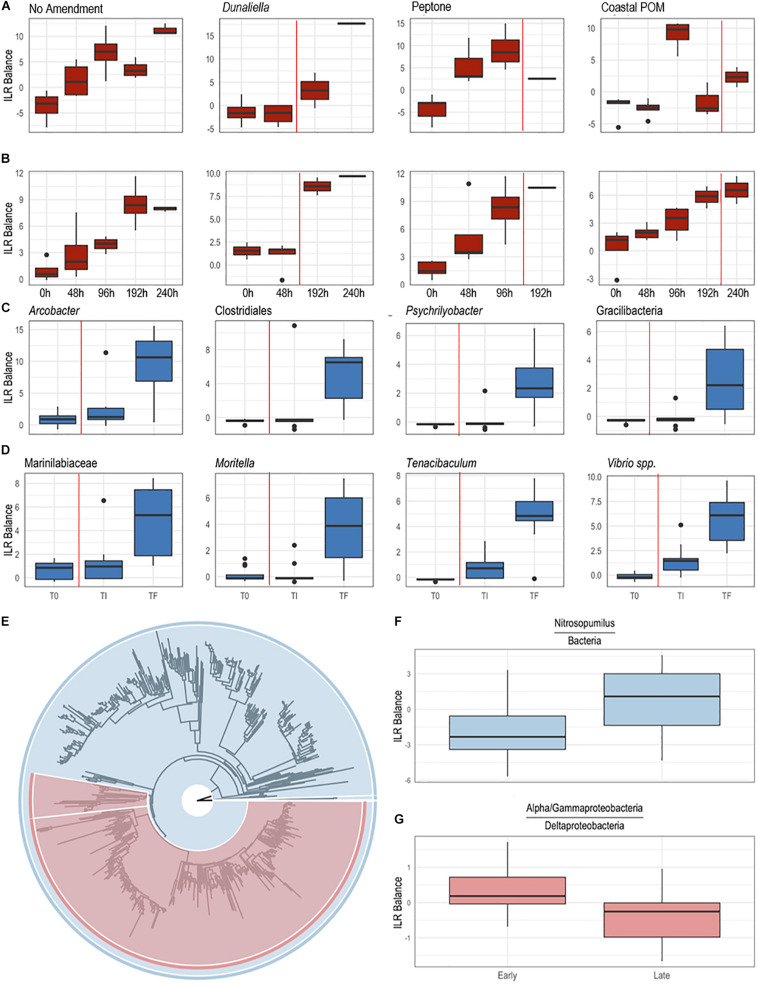
Differential abundance of bacterial taxa from surface swabs [**(A,B,E–G)**; *P. ochraceus* August 2019]; and body wall samples [**(C,D)**; *P. ochraceus* June 2018]. **(A,B)** were derived from a PhyloFactor object and show the ILR balance of Flavobacteriales **(A)** and Rhodobacterales **(B)** relative to all other sOTUs. Organic amendment is given above boxplots. Total sample numbers for each treatment (which varied due to the loss of asteroids over the course of the experiment to wasting) is given in Figure 4. The vertical red line in panels **(A–D)** indicate the average time at which asteroids formed lesions. **(C,D)** Boxplots were derived using PhyloFactor (Washburne et al., 2017), which uses a generalized linear model to regress the isometric log-ratio (ILR balance) between opposing clades (contrasted by an edge) on a phylogenetic tree. This was done iteratively, with each iteration, or factor, maximizing the *F* statistic from regression. Shown taxa represent either a single factor or combination of factors (when, for example, multiple factors identified different sOTUs with the same taxonomic classification). Labels represent either the highest taxonomic resolution or the highest classification shared by all sOTUs of a given clade. T0, experiment commencement; TI, lesion genesis; TF, time of death. **(E–G)** Balance contrast of early (before lesion genesis) samples compared to late (immediately prior to lesion genesis) samples. Samples were transformed using the Phylogenetic Isometric Log-Ratio (PhILR; Silverman et al., 2017) transform, which uses a phylogenetic tree **(E)** to convert an sOTU table into a new matrix of coordinates derived from the ILR of clades that descend from a common node. We used a sparse logistic regression with an *l*_1_ penalty of λ = 0.15 (Silverman et al., 2017) to analyze the ILR at each node, and included a select number of “balances” with positive coefficients **(F,G)**. **(F)** is the balance of *Nitrosopumilus* (colored blue in **(E)**, comprises the thin sliver on the right side of the tree) relative to the rest of the dataset [also shown in blue in **(E)**]. A positive shift indicates an increase in *Nitrosopumilus* relative to its denominator. **(G)** is the balance between a clade of Alpha/Gammaproteobacteria [large, red clade in **(E)**] and Deltaproteobacteria [Bdellovibrionales and Desulfobacterales; small, red clade in **(E)**]. A negative shift indicates that the denominator, Deltaproteobacteria, is increasing relative to Alpha/Gammaproteobacteria.

The boundary layer microbiota of *P. ochraceus* during the OM amendment experiment changed over time in all treatments (Figure 4 and Supplementary Figure S5), but the most prominent changes were distinguished by the copiotrophic orders Flavobacteriales and Rhodobacterales (Flavobacteriales; control: *p* < 0.001, *Dunaliella*: *p* = 0.002, peptone: *p* = 0.001, coastal POM: *p* < 0.001, ANOVA fit with a Generalized Linear Model) (Rhodobacteriales; control: *p* < 0.001, *Dunaliella*: *p* < 0.001, peptone: *p* = 0.010, coastal POM: *p* < 0.001, ANOVA with a Generalized Linear Model), which increased uniformly in all incubations (*n* = 5 per treatment), indicating that captivity alone may stimulate these groups (i.e., containment affect; Figures 5A,B). Unamended and peptone amended *P. ochraceus* experienced the most consistent change in Flavobacteriales, with both conditions exhibiting a linear increase in population mean relative to the mean of all other sub-OTUs. Flavobacteriales in coastal POM-supplemented *P. ochraceus* were elevated from the first to final timepoints, but were primarily distinguished by a large boom and bust after 96 h (c.f. bacterial abundance below; Figure 5A). This spike was due to an increase in the family *Crocinitomicaceae*, which, in addition to the family *Flavobacteriaceae*, comprised the majority of Flavobacteriales across all treatments. Rhodobacterales, which primarily consisted of the family *Rhodobacteraceae*, increased in all experimental conditions (Figure 5B).

### SSW in the Absence of External Stimuli

**FIGURE 6:**
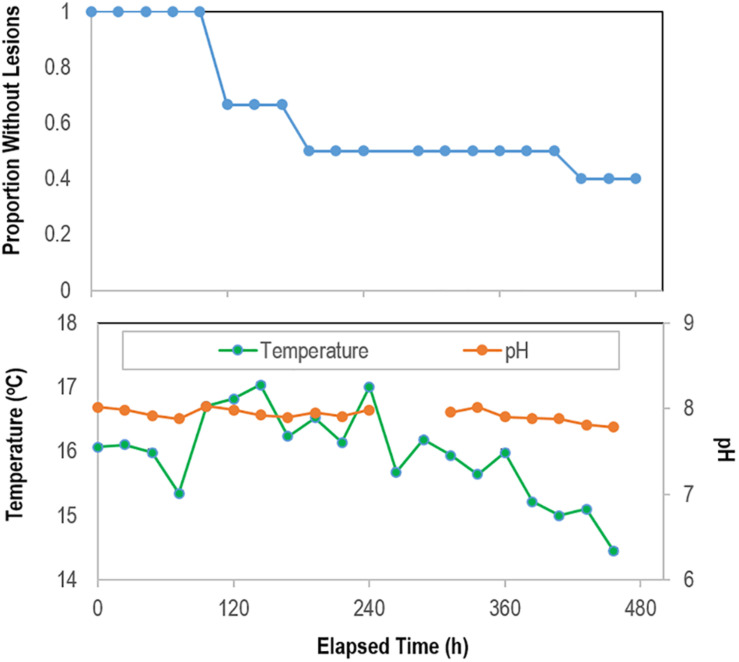
Proportion of grossly normal *P. ochraceus* (*n* = 6) remaining over time during longitudinal study of microbiome composition in the absence of external stimuli. The mean flow rate into aquariums was 3.81 ± 0.05 mL s^–1^ (average residence time in aquariums 37 min). The panel below shows temperature and pH during the experimental period.

In the August 2018 study, *Pisaster ochraceus* developed lesions without stimuli beginning 120 h after isolation, and by 192 h more than half of incubated specimens (*n* = 6) had SSW lesions (Figure 6). Lesions formed initially concomitant with an approx. 2°C swing in temperature, however continued in other specimens as temperatures progressively decreased over the course of the experiment. Lesions were grossly characterized by non-focal loss of epidermal tissues, which exposed underlying body wall tissues. Lesion margins were not melanized or otherwise discolored. Epidermal samples from these specimens revealed no significant difference in microbial composition between artificial and natural lesions in 3 specimens that wasted (PERMANOVA; *P* = 0.139; Supplementary Figure S6). Between initial samples and the time of lesion genesis, most taxa identified as differentially abundant were less abundant sub-OTUs.

In the May–June 2018 study, specimens had developed SSW lesions when observed after 312 h (10 of 12 specimens), and after 360 h all specimens has lesions. The time of death for all specimens occurred between 260 and 480 h. Because we did not capture the exact time of lesion occurrence, we restricted analysis of these individuals to samples collected at initial (T0), 360 h (TI—i.e., shortly after lesions had formed) and at time of death (TF). We observed a progressive increase in copiotrophic orders between initial samples and those taken shortly after lesions had formed, including Campylobacterales (*p* < 0.001), Flavobacteriales (*p* < 0.001), and Vibrionales (*p* < 0.001; ANOVA fit with a Generalized Linear Model) (Figures 5C,D). This occurred concomitant with an increase in *Nitrosopumilus* and obligate anaerobes (Deltaproteobacteria) relative to a large clade of typically fast-growing phyla (Alpha-and Gammaproteobacteria) (Figures 5E–G). Between lesion genesis and animal death, we observed a further increase in copiotrophs in body wall (May-June 2018) and epidermal (August 2018) samples, and a proliferation in microaerophiles (*Arcobacter* spp.), facultative anaerobes (*Moritella* spp.), and obligate anaerobic families (Clostridia, Fusobacteria and Bacteroidia) at time of death (Figures 5C,D and Supplementary Figure S7). The two sub-OTUs with the largest F-statistic from regression over the entire course of SSW from initial samples to animal death belonged to the families *Desulfobulbaceae* (*p* < 0.001) and *Desulfovibrionacea* (*p* = 0.002; ANOVA fit with a Generalized Linear Model).

### Impact of Depleted O_2_ Conditions on SSW in *Asterias forbesi*

**FIGURE 7:**
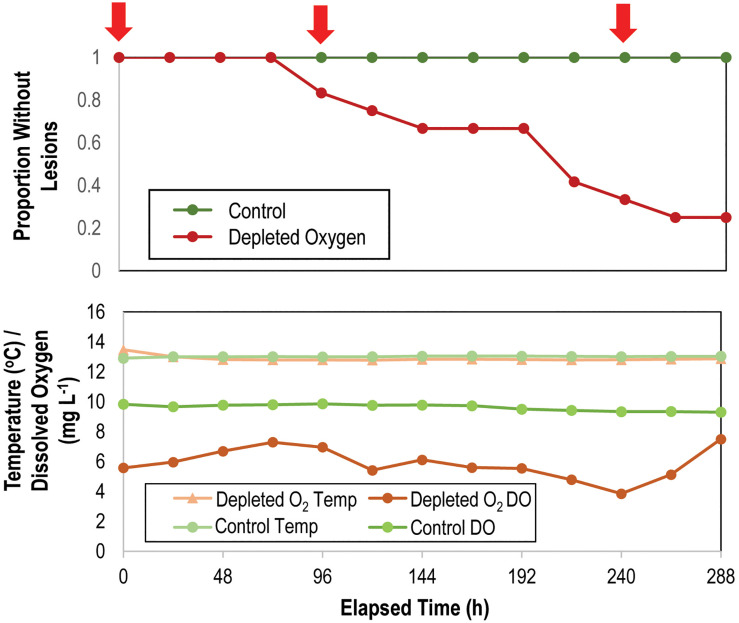
Proportion of grossly normal *Asterias forbesi* (*n* = 12 per treatment) incubated in normoxic and depleted oxygen water (top) and variation in temperature and O_2_ in incubation aquaria (bottom) over time. The red arrows at top indicate samples which were included in analysis of microbiome composition (see Supplementary Figure S6).

O_2_ was depleted in N_2_-sparged sump waters (5.87 ± 0.29 mgL^–1^) compared to control incubations (9.62 ± 0.06 mg L^–1^), representing a mean concentration decrease of ∼39% (Figure 7). All individuals remained grossly normal in control incubations over the 312 h experiment, while 75% of individuals in depleted O_2_ conditions developed lesions (mean time to lesion genesis = 9.58 ± 0.89 d; Figure 7). There was no significant difference in the rate of mass change between treatments, where all individuals lost on average 30.0 ± 6.5% of the initial mass over the course of the experiment as they were not fed (Supplementary Figure S3). Development of lesions over time was strongly related to treatment (*p* = 0.006, log-rank test, *df* = 12). Bacterial abundance on animal surfaces (which we define as abundance in surface samples) corrected for aquarium water values increased in both control and depleted O_2_ treatments over the first 144 h of incubation, but by 312 h, abundance of bacteria in depleted O_2_ treatments was significantly lower (*p* < 0.001, Student’s *t*-test, *df* = 12) on depleted O_2_ treated individuals than in control individuals (Supplementary Figure S8). *Asterias forbesi* treated with depleted O_2_ waters demonstrated consistent shifts in microbial communities with treatment (Supplementary Figure S6). However, no single bacterial taxonomic organization strongly differentiated normoxic from depleted O_2_ conditions.

### Experiments Examining Flow Rate, Desiccation, and Tissue Homogenate Challenge

**FIGURE 8:**
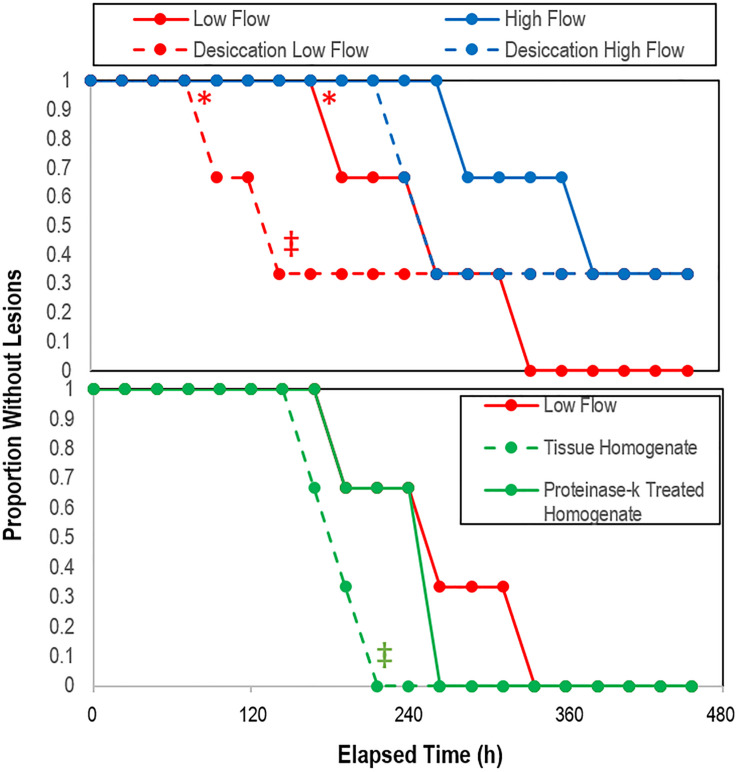
Proportion grossly normal *P. ochraceus* over time in response to treatment (top) desiccation (*n* = 3), and (bottom) treatment with crude and proteinase k-treated tissue homogenates (*n* = 2). * indicates that low flow lesion genesis time was significantly different (*p* < 0.05, Student’s *t*-test) to high flow rate; ‡ indicates that the overall trend of desiccation under low vs high flow rate and with the addition of proteinase-k treated homogenate vs. low flow controls was significant (*p* < 0.05, log-rank test).

All experiments were performed over a 504 h period during which mean water flow-through temperatures ranged from 14.4 to 17.6°C (Supplementary Figure S9), with day-night variation of 1.8–1.9°C. Water temperatures increased mostly between 240 and 360 h of incubation. Salinity did not vary by more than 0.2 over the course of the experiment. The time to lesion genesis in *Pisaster ochraceus* was faster for asteroids under low-flow conditions than those under high flow conditions (*p* = 0.006, Student’s *t*-test, *df* = 3), however survival was not significantly different between flows (log-rank test, ns; Supplementary Table S5). Desiccation resulted in faster lesion formation (*p* = 0.05, Student’s *t*-test, *df* = 3) under low flow conditions, but not different under high flow conditions (log-rank test, ns; Figure 8). Addition of tissue homogenates resulted in faster SSW than addition of proteinase k treated tissue homogenates (*p* = 0.04, Student’s *t*-test, *df* = 3); however, survival was no different between control treatment low flow and the addition of proteinase K-treated or untreated tissue homogenates (log rank test, ns; Figure 8).

### Modeling of SSW in Context of Experimental Parameters

Across all experiments, analysis of covariance revealed that lesion time was explained best by different parameters (Table 1). SSW lesion genesis rate across all experiments was significantly (*p* = 0.006; analysis of covariance) and positively related to initial animal mass. For *Asterias forbesi*, lesion genesis time variation was best explained by both overall animal mass change during the experiment and initial bacterial abundance (*p* = 0.12). In both the OM addition experiment alone and combining the results of OM addition experiments with flow rate experiments, lesion genesis time in *Pisaster ochraceus* was best explained by initial animal mass (*p* = 0.006). In OM addition, variation in lesion genesis time was also explained by change in bacterial abundance over the first 72 h of incubation (*p* = 0.018).

**TABLE 1:** Multiple linear regression models of lesion time against measured experimental parameters.

Experiment	Variable 1	Variable 2	Model	*p*
*Asterias forbesi*—Oxygen	MC (*p* = 0.025)	IB (*p* = 0.036)	LT = 33.78−3.99*IB + 42.49*MC	0.012
*Pisaster ochraceus*—Flow Rate	FR (*p* = 0.025)	−	LT = 19.46−0.01*AM-0.22*FR	0.004
*Pisaster ochraceus*—OM	AM (*p* = 0.011)	TB (*p* = 0.031)	LT = 12.86−0.01*AM-151.72*TB	0.018
*Pisaster ochraceus* Flow Rate + OM	AM (*p* = 0.0017)	−	LT = 13.98−0.02*AM-0.28*MC	0.006

*LT, Lesion Time; MC, Mass change over experiment; IB, Initial bacterial abundance; FR, Flow Rate; AM, Animal Mass at Experiment Initiation; TB, Bacterial abundance change over first 3 days of experiment. Models were performed using stepwise (forward selection) regression with change in Akaike’s AIC as entry criteria.*

### Comparison of Asteroid Volume and Rugosity With SSW Susceptibility

**FIGURE 9:**
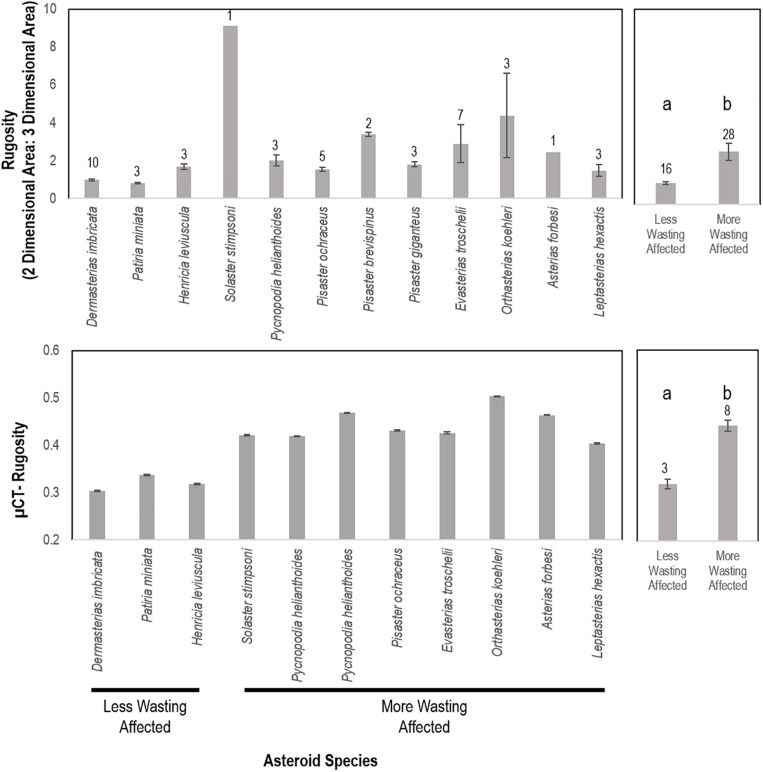
Rugosity of similarly sized specimens between wasting-affected and less wasting affected species as determined by whole animal computed tomography (top) and of an asteroid ray by micro-computed tomography (bottom). a,b denote significant difference at *p* < 0.001. More and less wasting affected assignment were based upon previous work and defined in the text.

CT-derived volume was significantly and positively correlated with overall mass across all specimens (*p* = 0.00001, *R*^2^ = 0.9999). Surface area to ray length followed an exponential function [mean ray length = 0.0825e^1.0536^*LOG(Surface area)^, *R*^2^ = 0.93. Log (Surface area)] was significantly and linearly correlated with Log [Volume; LOG(Volume) = 0.7319^∗^LOG(Surface Area) + 0.7662; *R*^2^ = 0.9703] (Supplementary Figure S10). The surface area: volume was significantly and negatively correlated with a logarithmic function defined as SA:Vol = 4.6242e^–0^.^549[LOG(Volume)]^. The mean rugosity (defined as 3D:2D surface area as measured by CT) was significantly (*p* = 0.015, Student’s *t*-test, *df* = 14) greater in less affected species than more affected species (Figure 9). Surface area:volume, individual specimen mass, and overall surface area were not significantly different between categories among similarly sized animals. The rugosity of SSW-affected taxa as measured by micro-CT was significantly (*p* = 0.0002, Student’s *t*-test, *df* = 4) greater than less-SSW affected species (Figure 9).

### Comparison of Respiration Rates Between Species

Mass-normalized measured asteroid respiration rates of asteroids were greatest for *Asterias forbesi*, and least for *Dermasterias imbricata* and *Patiria miniata* (Supplementary Table S6). Both *Pisaster ochraceus* and *Asterias forbesi* respiration rates were considerably more than for other specimens. The ratios of measured respiration rate to theoretical maximum diffusion rates into coelomic fluids were greatest in *Asterias forbesi* and *Pisaster ochraceus* (which were both > 1 in most specimens) and least in *Patiria miniata* and *Dermasterias imbricata* (which were always < 0.1).

### Analysis of SSW Occurrence in Context of Environmental Parameters

**FIGURE 10:**
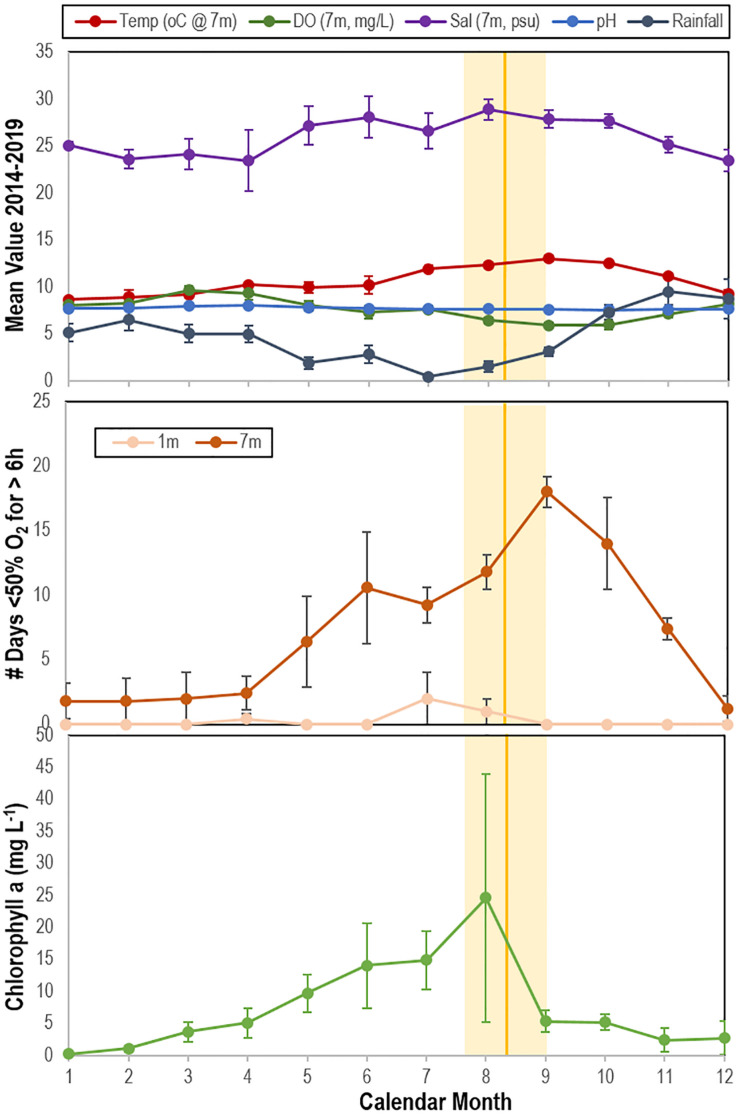
Correspondence between mean time of wasting mass mortality (indicated by solid orange line (SE range indicated by lighter orange bar) compared with physico-chemical parameters (top), the mean number of days in which oxygen was < 50% saturation for ≥ 6 h at 1 and 7 m (middle) and mean chlorophyll a concentration (bottom) at Penn Cove, Whidbey Island from 2014 to 2019. Temp, temperature; DO, dissolved oxygen; Sal, salinity. Data was analyzed from the Penn Cove Shellfish data buoy (retrieved from http://nvs.nanoos.org).

The mean time of SSW mass mortality observed at Whidbey Island between 2014 and 2019 fell at or within 1 month after the mean annual maximum of chlorophyll a and minimum rainfall (Figure 10). The mean time of SSW mass mortality fell immediately prior to the maximum frequency of transient water column depleted O_2_ events (i.e., days per month where O_2_ was < 50% saturation for > 6 h) (Figure 10). Multiple linear regression (stepwise, backward selection criteria) revealed a significant model (*R*^2^ = 0.866; *p* = 0.001) where temperature (*p* = 0.006), chlorophyll a (*p* = 0.027) and salinity (*p* = 0.044) explained most variation in SSW mass mortality, while forward selection (*R*^2^ = 0.774; *p* = 0.0002) revealed that monthly variation in SSW was significantly explained by O_2_ alone. Mass mortality was significantly related (one-way ANOVA, *p* < 0.0001) to elevated chlorophyll in the previous 3 months relative to non-mass mortality months, to elevated salinity, and reduced rainfall (Supplementary Figure S11).

### Stable Isotopic Comparison of SSW-Affected and Grossly Normal Specimens

**FIGURE 11:**
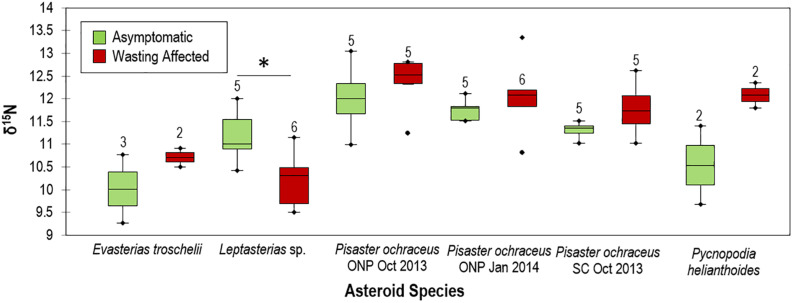
Comparison of grossly normal and wasting δ^15^N values between species. ONP = Starfish point, Olympic national park and SC = Davenport, Santa Cruz, CA. * indicates *p* < 0.05. Numbers above box plots indicate *n* of specimens used in comparison.

The natural abundance of ^15^N (δ^15^N) and δ^13^C varied between species, with highest values for *Hippasteria spinosa* and lowest for *Pteraster tesselatus* (Supplementary Figure S12). There was no correspondence between known diet of asteroids (and, hence, food web position) and relative stable isotope composition between species. SSW-affected asteroids (including *Pisaster ochraceus*, *Pycnopodia helianthoides*, and *Evasterias troschelii*), had generally higher δ^15^N in their tissues than grossly normal tissues at the same site within-species (ns) except for *Leptasterias* sp., which had significantly lower δ^15^N in SSW-affected tissues than in grossly normal individuals (Figure 11). On average, δ^15^N was enriched by 3.9 ± 3.3% for each species (7.0 ± 2.8% excluding *Leptasterias* sp.) in SSW-affected compared to grossly normal specimens. Ellipse analysis, which can be used to infer isotopic niches or metabolic differences between populations (Jackson et al., 2011) suggested that in all paired site-species comparisons SSW-affected specimens have altered C and N metabolisms compared to grossly normal individuals (Supplementary Figure S13).

## Discussion

The development of SSW lesions under depleted O_2_ conditions and increase in anaerobes on asteroid surfaces during SSW progression suggest that anaerobic environments likely form in response to OM input. Our work also suggests that this OM input may be related to primary production, since SSW correlated with chlorophyll a trends at a field site and amendment with algal-derived OM resulted in faster wasting than controls. Furthermore, our results suggest that this interaction may have occurred during mass mortality in 2013–2014, since δ^15^N was enriched in affected tissues relative to grossly normal individuals collected at the time. Collectively, the results of this study support the hypothesis that SSW is influenced by microbial activities at the animal-water interface, which may result in oxygen diffusion limitation within the DBL per our model (Figure 1).

### OM Amendment Stimulates Boundary Layer Microorganisms and Results in Rapid SSW

The impact of elevated heterotrophic bacterial respiration on animal surfaces was studied through amendment with various sources of OM which we hypothesized would fuel microbial remineralization. These data show that amendment with some OM substrates—a frozen (at −20°C) and thawed unialgal phytoplankton culture (*Dunaliella teriolecta*) and peptone, which is frequently used in marine bacterial culture media—resulted in faster lesion genesis than untreated specimens. Bacterial abundance increased prior to lesion genesis in at least two OM treatments, which suggests that our OM amendment strategy was successful in stimulating bacterial growth, and may indicate the proliferation of copiotrophic taxa. The subsequent decrease in bacterial abundance after initial increase in both peptone, coastal POM, and consistently lower bacterial abundance in *Dunaliella*-POM incubations may be evidence of heterotrophic remineralization-fueled O_2_ deficit over time on SSW-affected asteroids, similar to the effect observed in our experiments with *Asterias forbesi* incubated in depleted O_2_ water (see below). Facultative and strict anaerobes generally experience slow growth rates compared to aerobic taxa because it is less energetically efficient to grow on reduced electron acceptors. While standing stock of aquatic bacteria may be higher in anaerobic conditions than in aerobic conditions, population growth rates are typically lower (Cole and Pace, 1995).

### SSW Is Induced by Depleted O_2_ Conditions

Our data demonstrate that SSW is induced by depleted O_2_ water column conditions. We incubated *A. forbesi* in depleted O_2_ water and observed patterns of SSW progression, boundary layer bacterial abundance and microbial assemblage β-diversity. Dissolved O_2_ (DO) concentrations were controlled in an aquarium setting by continuous sparging with N_2_, which were on average 39% lower than untreated control incubations (Figure 7). Despite the small decrease in DO in experimental incubations, which was well above the threshold for hypoxia (∼80% decrease in DO compared to saturation), we observed significant impacts on SSW progression and microorganisms inhabiting the animal-water interface. SSW lesions were only observed in depleted O_2_ water treated specimens, which were accompanied by an initial increase in bacterial abundance followed by lower bacterial abundance after lesions had developed, which is a result that is similar to observed trends during OM enrichment. While microbial communities progressively changed during the experiment, high variation between replicate specimens precluded identification of any taxonomic organizational unit associated with treatment.

Further evidence for the role of O_2_ in SSW was observed in experiments comparing lesion genesis speed under variable incubation flow rates (i.e., water replenishment rates) which found faster lesion genesis under low flow conditions when compared to high flow conditions, and in the desiccation experiment, (which was used to insult asteroids and simulate emersion during low tide events in warmer temperatures) which also resulted in faster lesion formation under low flow conditions, but not in high flow conditions. While we did not measure DO concentrations in these experiments, higher flow rates likely had higher DO concentrations than lower flow rates, in addition to reducing OM (e.g., mucus) and toxic exudates in animal waste [notably NH_3_ (Propp et al., 1983) and S^–^ (Vistisen and Vismann, 1997)]. Furthermore, higher flow rates may have experienced less extensive boundary layers than lower flow rates (Fonseca and Kenworthy, 1987).

### Shifts in Heterotrophic Bacterial and Archaeal Communities During SSW Progression in Experiments and in the Absence of External Stimuli

We surveyed the composition of bacterial and archaeal communities in experiments and in the absence of external stimuli to gain insight into their environmental conditions. Because sampling by biopsy punch imparts stress on animals that may elicit SSW, sampling in the absence of external stimuli focused on samples collected at the time of lesion genesis (August 2018) and samples collected after lesion genesis (May–June 2018), and are distinct from samples collected by surface swab prior to lesion genesis (collected in OM enrichment and depleted O_2_ water experiments).

Prior to lesion genesis we observed a general proliferation of well-known copiotrophic marine bacterial orders (Flavobacteriales and Rhodobacteriales) in both controls and OM amendments. Several studies have observed the proliferation of copiotrophic taxa longitudinally during SSW, including genera within the families *Flavobacteriaceae*, *Rhodobacteriacaea* (Lloyd and Pespeni, 2018), Actinobacteria, and genera in the orders Altermonadales (Nuñez-Pons et al., 2018), Vibrionales and Oceanospiralles (Hoj et al., 2018). These taxonomic groups are amongst the most active constituents of bacterioplankton and major players in marine OM degradation, some of which have facultative anaerobic metabolisms (Pinhassi et al., 2004; Choi et al., 2010; Buchan et al., 2014; Thiele et al., 2017; Pohlner et al., 2019). While it is tempting to ascribe pathogenicity traits to groups that are enriched on disease-affected tissues (based on members of the same family or genus causing pathology), or infer their role in community dysbiosis (i.e., the microbial boundary effect), this is not possible in the absence of demonstrated pathogenicity or strain-level assignment (Hewson, 2019).

At the time of, and following lesion formation, bacteria on surfaces likely experience a complex milieu of OM molecules, including those from decaying tissues. We found evidence for further increases in copiotrophic taxa, and the proliferation of microaerophiles (*Arcobacter* spp.), facultatively anaerobes (*Moritella* spp.), and obligate anaerobic classes (Clostridia, Fusobacter, Bacteroidea) and families (*Desulfobulbaceae* and *Desulfurovibrionaceae*), likely remineralizing dead asteroid tissue-derived substrates, between lesion formation and animal death in/on epidermal tissues of asteroids that wasted in the absence of external stimuli. These results are consistent with the pattern of microbial assemblage variation observed in the OM amendment experiment. Previous study comparing SSW-affected (specimens already had lesions) and grossly normal asteroid-associated community gene transcription also noted the increase in transcripts from *Propionibacterium*, *Lachnospiraceae*, and *Methanosarcina*, which are strict anaerobes, as well as *Stigmatella* and *Staphylococcus*, which are facultative anaerobes, as a proportion of total transcripts (Gudenkauf and Hewson, 2015).

Bacterial stimulation and SSW in asteroids is paralleled by the DDAM (dissolved organic carbon, disease, algae, microorganism) positive feedback loop in tropical corals (Dinsdale et al., 2008; Barott and Rohwer, 2012; Silveira et al., 2019). Coral disease is associated with OM cenrichment (Kline et al., 2006; Smith et al., 2006), some of which originates from sympatric primary producers (Haas et al., 2010, 2011), which in turn are more labile than OM released from the corals themselves (Haas et al., 2016; Nakajima et al., 2018) and results in both elevated bacterial abundance on coral surfaces (Dinsdale and Rohwer, 2011; Haas et al., 2016), and enhanced remineralization rates (Haas et al., 2016). In black band disease, DOC released from primary production causes micro-zones of hypoxia which result in production of toxic sulfides, which in turn result in opening of niches for cyanobacteria (Sato et al., 2017). One possible mechanism by which bacterial proliferation affects asteroid tissues is through the secretion of bacterial toxins and other virulence factors (e.g., proteases) which directly damage tissues and may contribute to lesion development. For example, *Vibrio coralliilyticus* induces coral tissue damage by excretion of a metalloproteases (Santos et al., 2011). Release of secreted enzymes more generally by a wider suite of copiotrophic taxa may react with asteroid tissues and generate non-specific immune responses. Anaerobic conditions may cause upregulation of virulence factor gene expression, or they may be upregulated in response to quorum sensing during proliferation. In SSW, the proliferation of heterotrophic bacteria and development of SSW may be due to any of these effects.

### SSW Is Related to Inherent Asteroid Properties That Dictate Boundary Layer Extent, Gas Diffusion, and Respiration

We hypothesized that SSW susceptibility may relate to both inter-species variation in rugosity (i.e., degree of corrugation), which dictates DBL thickness, and intra-species surface area-to-volume ratio, which determines total gas flux potential. Mean and turbulent flow structure around aquatic animals and plants relates to the mean height, density and shape of structures as they compare to flat surfaces (Koch, 1994; Nepf, 2011; Brodersen et al., 2015). We speculate that more extensive boundary layers may result in a greater deficit in O_2_ due to entrapment of OM adjacent to animal tissues, and also increase the potential for depleted O_2_ at the animal surface since the effective distance over which O_2_ must diffuse is higher in specimens with greater boundary layer extent. Direct measurement of O_2_ concentration in boundary layers as they relate to bacterial remineralization are precluded by the sensitivity of instruments (e.g., microelectrodes) to physical damage in non-immobilized specimens. Our results show that inter-species SSW susceptibility was related to specimen rugosity. This supports the idea that more rugose species may be more susceptible to SSW because of their greater DBL extent (physical distance) on respiratory surfaces.

SSW risk susceptibility may furthermore result from differential diffusive flux potential compared to respiratory demand. We found that the ratio of measured respiration rate to diffusive flux potential (RR:TD) was greatest in two asteroid species that were more affected by SSW and least in two species that were less affected. Perturbation of O_2_ availability in animal surface boundary layers may skew diffusive flux by elongating diffusive path length or reducing differences in O_2_ between tissues and surrounding seawater. Hence, specimens with a higher RR:TD may be more affected by the condition than those with lower RR:TD. We cannot account for variable permeability of outer epidermis between individuals (not measured), and assume that all surface area of asteroids is involved in respiration (which may be over-estimated, since presumably some component of this area comprises mineral structures).

### Potential Sources of OM Fueling Microbial Activity

Heterotrophic bacteria in marine environments remineralize OM that originates from autochthonous and allochthonous sources (Ducklow, 1983; Benner et al., 1992; Amon and Benner, 1996). We hypothesized that there are two primary sources fueling microbial activity: OM from primary production, and OM from decaying asteroids. Most SSW is reported in late fall or summer, with fewer reports during other times of the year (Eckert et al., 1999; Bates et al., 2009; Eisenlord et al., 2016; Menge et al., 2016; Montecino-Latorre et al., 2016; Hewson et al., 2018, 2019; Miner et al., 2018; Harvell et al., 2019). Among the myriad of OM sources in seawater, phytoplankton-derived OM is highly labile (Ochiai et al., 1980; Ogawa and Tanoue, 2003; Thornton, 2014). We observed correspondence between the occurrence of mass mortality at or after the mean annual maximum chlorophyll a concentration and minimum rainfall, where temperature, chlorophyll a and salinity explained most variation in asteroid mass mortality. Because SSW occurs seasonally in late summer and early autumn, and there is therefore autocorrelation between these environmental conditions, correlation between mass mortality and these parameters alone does not necessarily indicate a direct link between primary production and SSW. However, we also observed that asteroids challenged with POM from a phytoplankton (*Dunalella tertiolecta*) culture formed lesions faster than unamended controls, so it is possible that SSW is related to phytoplankton-derived DOM. A high pCO_2_ but low temperature-SSW positive relationship noted in Oregon may suggest that upwelling stimulated primary production at this location (Menge et al., 2016). The CALCoFi program observed highest coastal upwelling on record in 2013 in central California during SSW onset (Leising et al., 2014). These observations suggest that primary production intensity and timing in 2013–2014 departed from inter-annual variation in prior years, and has followed seasonal patterns since 2014.

OM originating from decaying asteroids may also contribute to microbial activity at the animal-water interface. Experimental challenge with asteroid tissue homogenates in this study (Figure 8), and reported previously (Hewson et al., 2014; Bucci et al., 2017), suggest that SSW may also be associated with decomposition of nearby asteroid individuals via assimilation of tissue-derived compounds and subsequently increased boundary layer microbial activity. Enrichment of near-benthic OM pools by SSW-affected individuals may have resulted in the apparent density dependence of SSW observed in 2014 in some populations (Hewson et al., 2014). Indeed, challenge with tissue homogenates by direct injection into coelomic cavities likely enriches within-and near animal OM pools, which in turn may stimulate heterotrophic remineralization. Hence, challenge experiments resulting in SSW, such as those performed previously (Hewson et al., 2014; Bucci et al., 2017) and in this study, may be a consequence of microbial activity induced by OM availability (and possibly protein-bearing material). The apparent transmissibility of SSW in field sites is based on observations of density dependence at some sites, along with geographic spread between adjacent sites and through public aquaria intake pipes (Hewson et al., 2014). These observations may be inaccurately ascribed to transmissible pathogenic microorganisms, since they may also be explained by enrichment of surrounding habitats and through intake pipes of OM pools from decaying individuals.

### Wasted Asteroids in 2013–2017 Bore Stable Isotopic Signatures of Anaerobic Processes

Because SSW has no pathognomic signs and has been reported for over a century (reviewed in Hewson et al., 2019), an obvious question is whether surface microbial activity was related to asteroid mass mortality observed from 2013. While retrospective analyses of O_2_ status of asteroids during this event is not possible, hypoxic conditions impart elemental signatures in tissues of preserved specimens. The elemental composition of asteroids, like all animals, largely reflects nutritional source, who obtain anabolic material from consumed prey. Furthermore, asteroids may take up DOM directly from the water column and use these materials for soft body parts, like tube feet (Ferguson, 1967a,b). The half-life of isotopic signatures in tissues relates to tissue turnover and is most stable in ectotherms (Vander Zanden et al., 2015). Dissimilatory anaerobic nitrogen cycling processes, such as denitrification, shift the balance between ^15^N and ^14^N (i.e., selecting against ^15^N), resulting in higher δ^15^N (ratio of tissue ^15^N to atmospheric ^15^N) in environments (Miyake and Wada, 1967). We examined δ^15^N in fast-growing, regenerative tube feet which reflected the most recent environmental conditions prior to collection, and compared its natural abundance between SSW-affected and grossly normal individuals collected at the same location and time, in 2013 and 2014. Our finding of enriched δ^15^N in tissues from SSW-affected specimens compared to grossly normal specimens suggests that SSW is associated with enhanced anaerobic dissimilatory respiration of nitrogen species in their environment, which can only occur in hypoxic or anaerobic conditions.

## Conclusion

Our results support the hypothesis that SSW is influenced by microorganisms inhabiting the animal-water interface. Elevated microbial activity due to enhanced OM supply may exacerbate SSW development. While the potential sources of OM fueling microbial activities in the DBL are numerous, we illustrate that both algal-derived OM and asteroid tissue-derived OM may precipitate SSW. Furthermore, the timing of wasting at a field site corresponds best with seasonal patterns of primary production and DO. The effects of elevated microbial activities within the asteroid DBL may be exacerbated under warmer ocean conditions, or conditions in which labile OM from terrestrial sources (which may include anthropogenic nutrient pollution) may be present in coastal environments.

## Data Availability Statement

The datasets presented in this study can be found in online repositories. The names of the repository/repositories and accession number(s) can be found below: https://www.ncbi.nlm.nih.gov/bioproject/PRJNA637333, Morphosource link at: https://www.morphosource.org/ sample identifiers S32007, S32014, S32018, S32021, S32022, S32029, S32031, S32036, S32037, S32039, S32042, S32046, S32047, S32048 and S32050, and https://qiita.ucsd.edu/ studies 12131 and 13061.

## Author Contributions

LS and IH designed the research. CA, RB, CD, JK, IP, PR, JR, LS, JS, and IH performed the research. CA, RB, CD, IP, PR, JR, LS, JS, JW, and IH conducted the review and wrote the manuscript. PR and IH provided funding for the research. All authors contributed to the article and approved the submitted version.

## Conflict of Interest

The authors declare that the research was conducted in the absence of any commercial or financial relationships that could be construed as a potential conflict of interest.

## Footnotes

1 http://nvs.nanoos.org/
