# Supplementary material for: Evidence That Microorganisms at the Animal-Water Interface Drive Sea Star Wasting Disease
Source: Front Microbiol. 2021 Jan 6;11:610009. doi: 10.3389/fmicb.2020.610009 (PMC7815596; doi:10.3389/fmicb.2020.610009)
Supplement: Supplementary file 1 [file Data_Sheet_1.pdf]

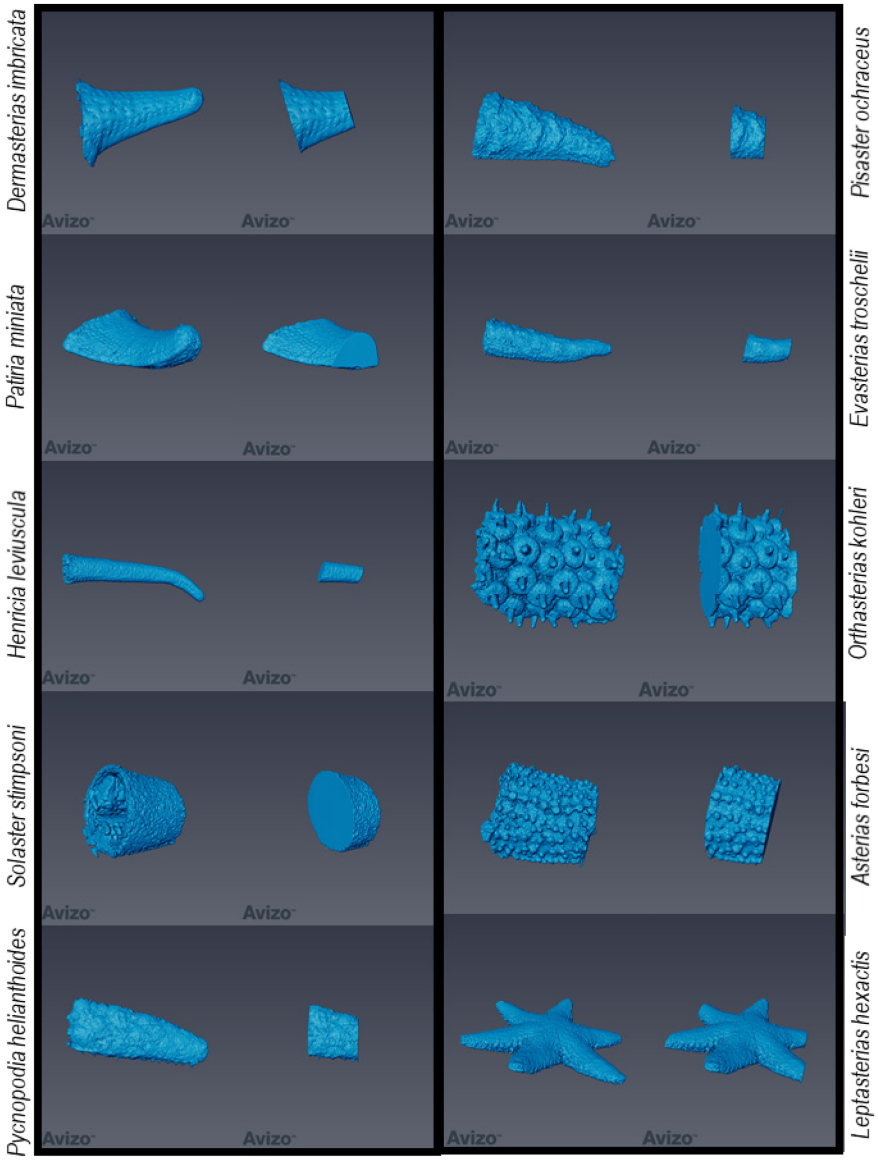

Fig. S1: Micro-computed tomographic images of asteroid rays used to examine rugosity. For each species, the entire ray (left) and the 1cm slice of the ray (right) is depicted.

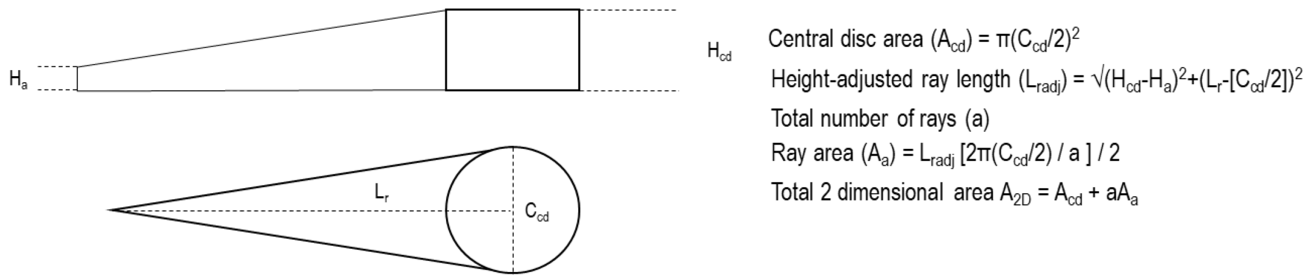

Fig. S2: Calculation of two dimensional area from parameters measured on starfish. Three dimensional area was determined by computed tomography.

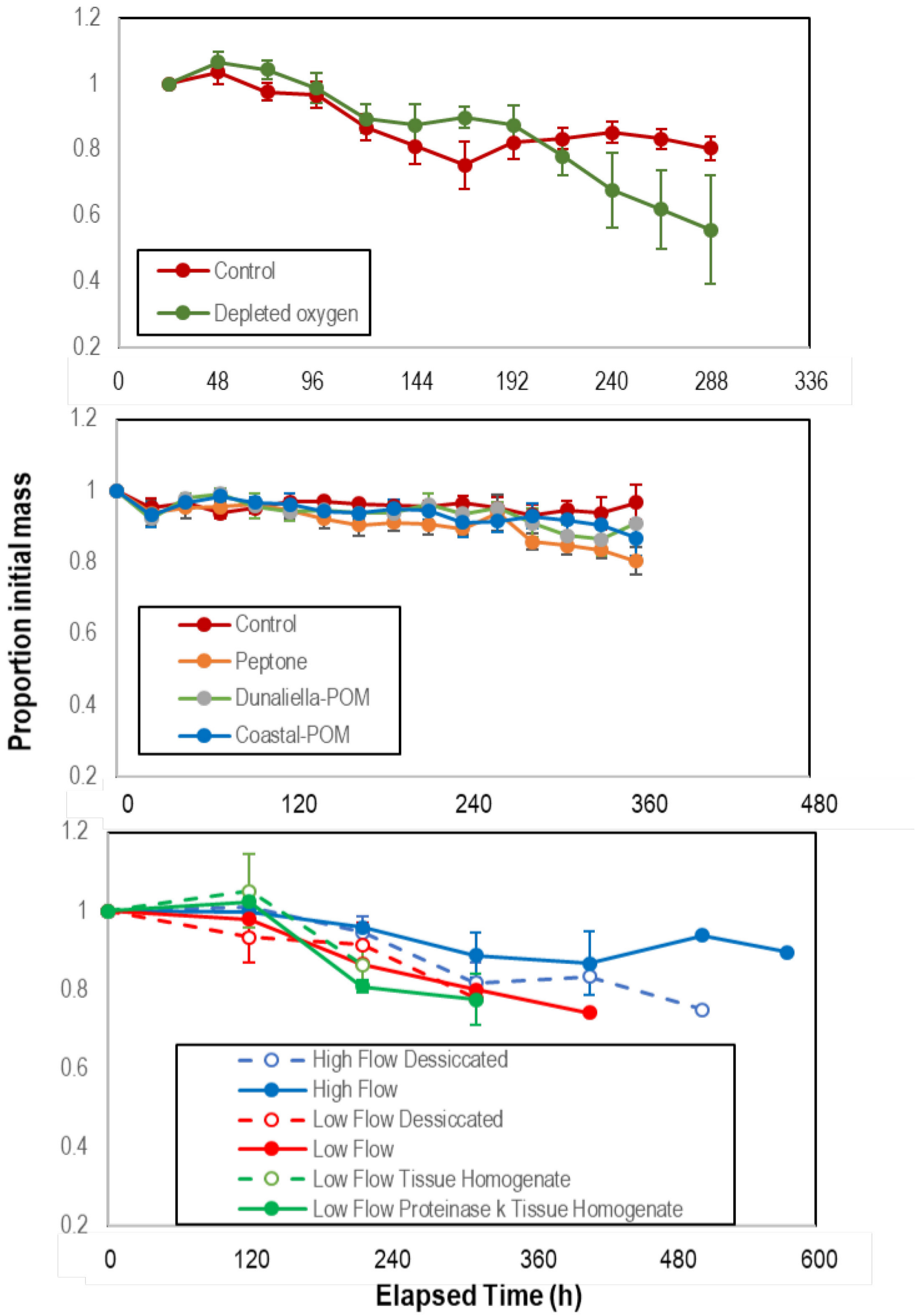

Fig. S3: Proportion original mass of individuals exposed to treatments with depleted O_2 (top), organic substrates (middle) and flow rates/desiccation and tissue homogenates (bottom) during challenge experiments. There was no significant difference in the rate of mass loss in any treatment within each experiment.

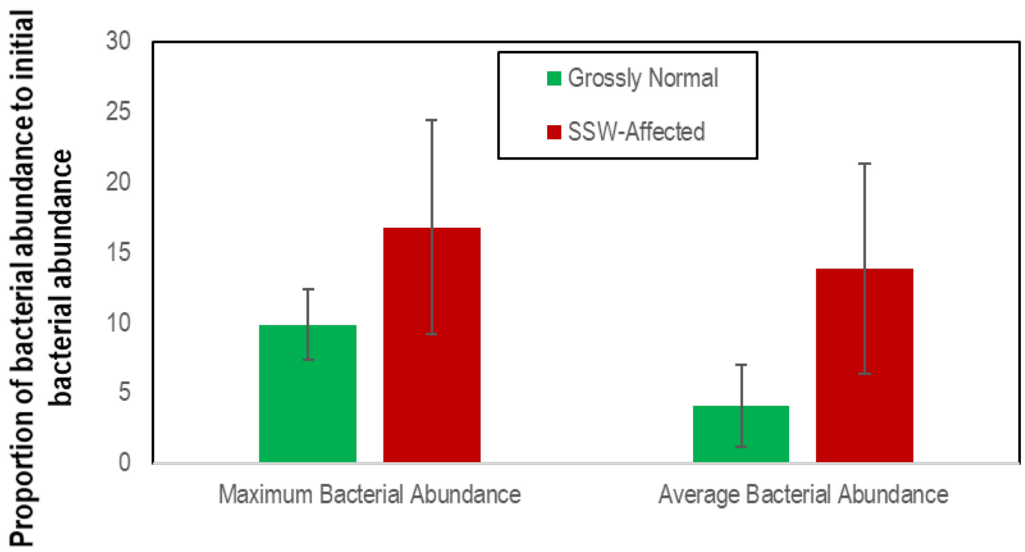

Fig. S4: Mean (±SE) proportion of maximum and average bacterial abundance to initial abundance on non-wasted stars and wasting-affected stars (regardless of treatment) during organic matter amendment experiment.

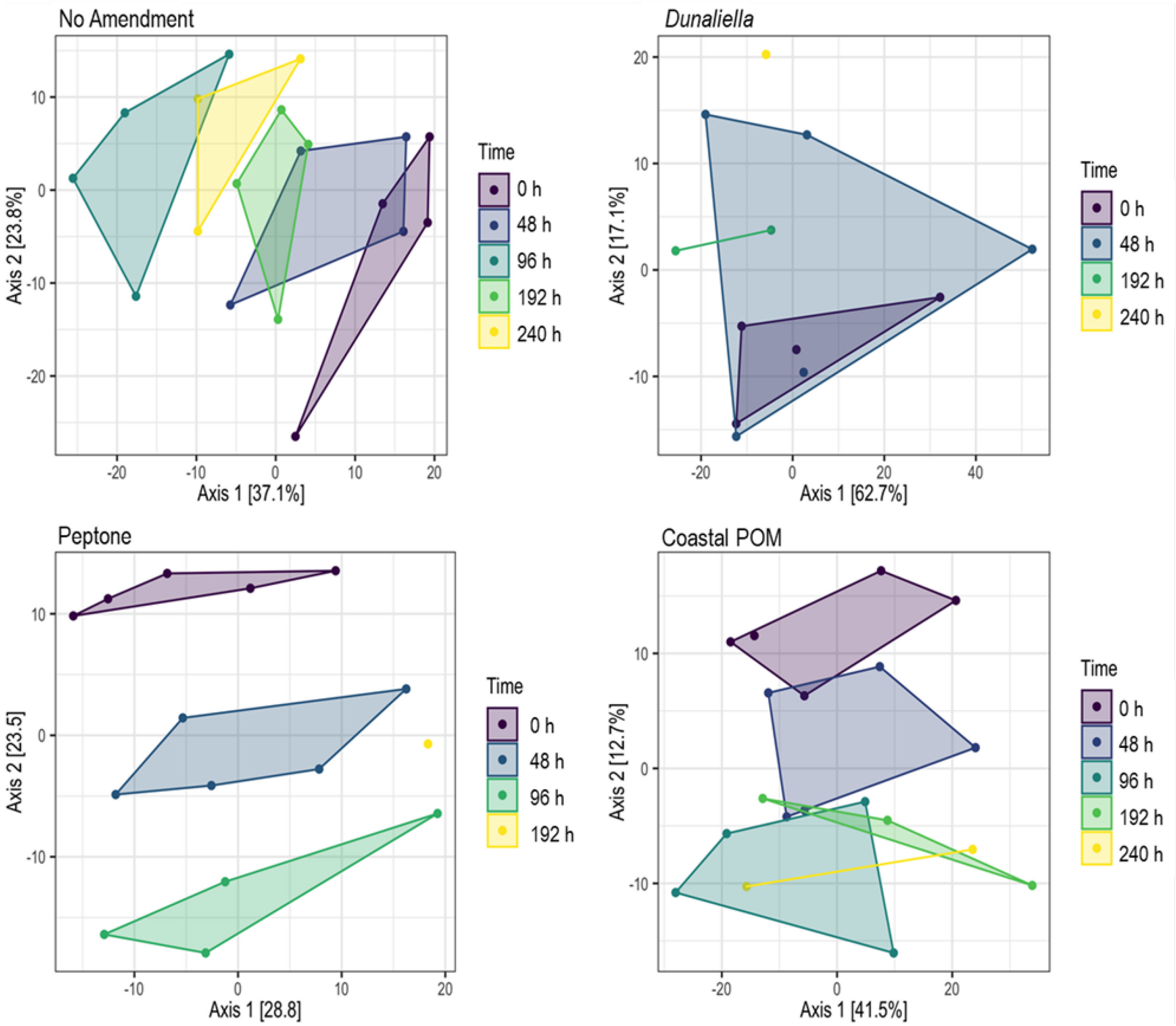

Fig. S5: Principal coordinate analyses (PCoA) of Euclidean distances after PhILR transformation. Bacterial communities were derived from surface swabs of *P. ochraceus* specimens enriched with the indicated organic material. Samples were taken over a timecourse indicated in the PCoA legends. Time 0 = initial samples; Time 2 = 48 h, Time 4 = 96 h, Time 8 = 192 h and Time 10 = 240 h. Organic amendment revealed much more variation before lesions first appeared than in body wall or epidermal samples taken at the time of lesion formation or afterwards.

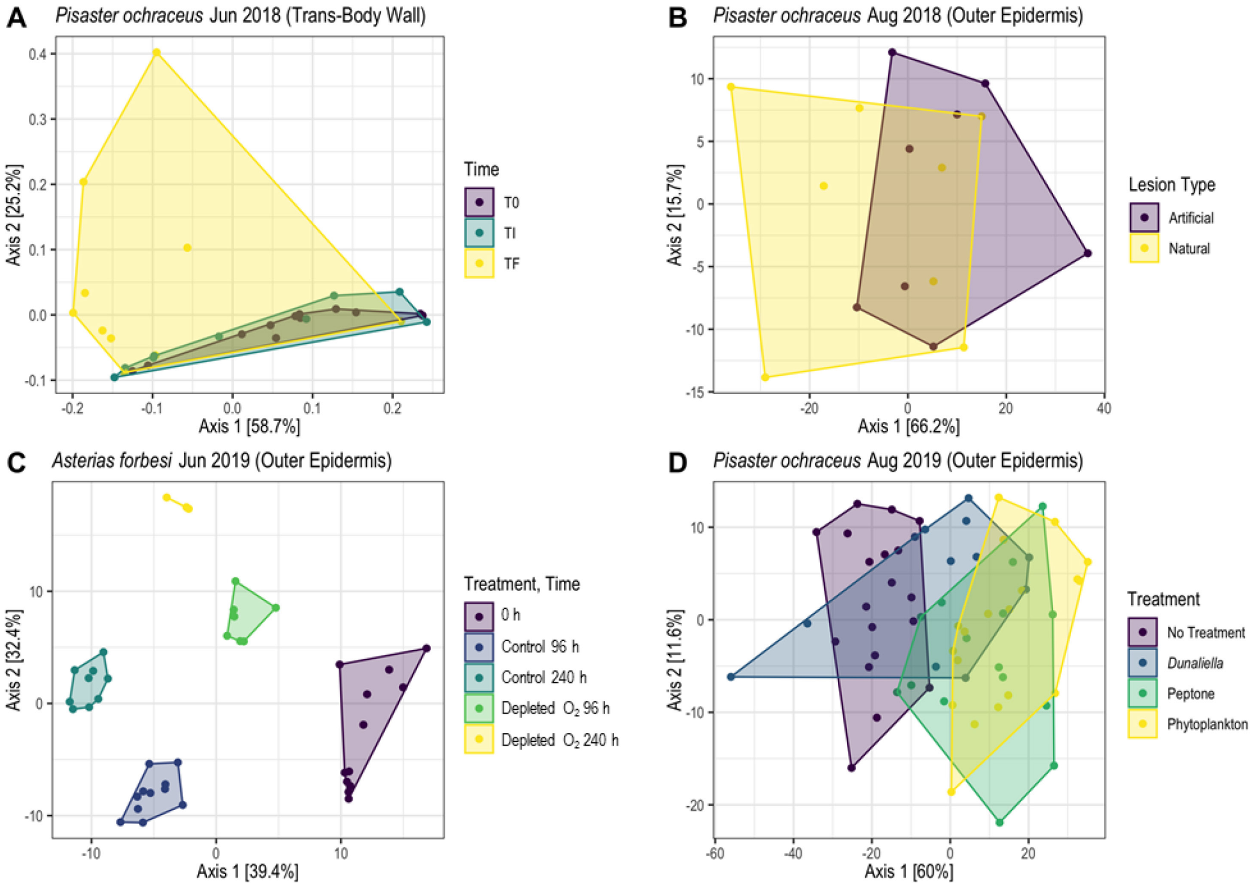

Fig S6: Principal coordinate analyses (PCoA) of bacterial communities using Weighted Unifrac distances (A) and Euclidean distances after PhILR transformation (B-C). (A) Body wall samples over the course of wasting. T0 = experiment commencement; TI = lesion genesis; TF = time of death. (B) Epidermal scrapes from artificially induced and naturally occurring lesions. (C) Surface swabs from asteroids under normoxic (control) and depleted O_2 conditions. T0 = experiment commencement; encompasses both normoxic and depleted O_2 conditions. T4, T10 = 96 hand 240 h, respectively. (D) Surface swabs from asteroids amended with different types of organic matter. The timecourse delineation for each treatment is shown in Fig. S5. In the May-June 2018 experiment, microbial assemblages were different between initial samples, after lesions had formed, and at the time of death in body wall samples (Principal Coordinate Analysis of Weighted Unifrac distances; PERMANOVA; P = 0.002).

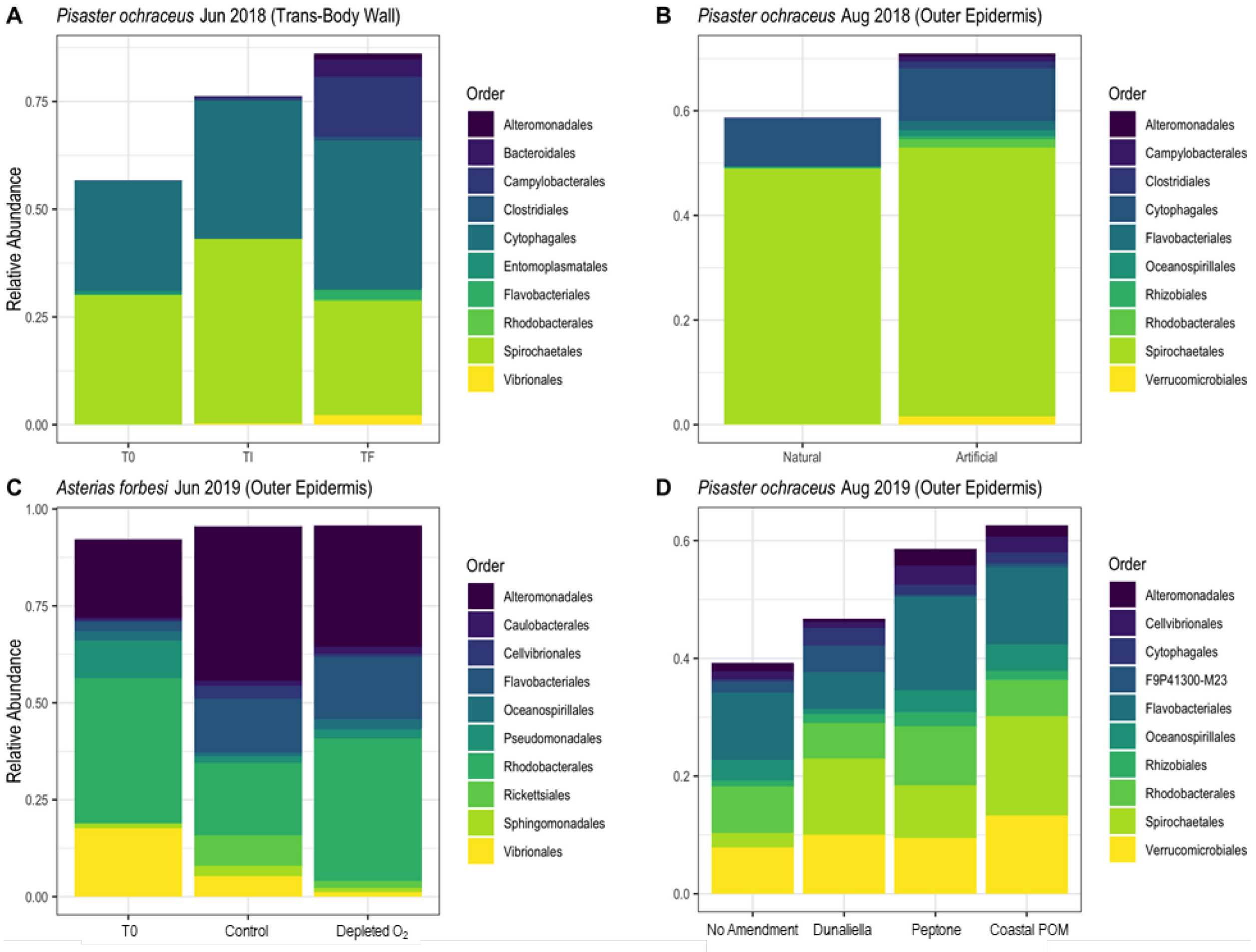

Fig S7: Relative abundances of bacterial orders across experiments. (A) Body wall samples over the course of wasting. T0 = experiment commencement; TI = lesion genesis; TF = time of death. (B) Epidermal scrapes from artificially induced and naturally occurring lesions. (C) Surface swabs from asteroids under normoxic (control) and depleted O_2 conditions. T0 represents all asteroids in both conditions at the beginning of the experiment. (D) Surface swabs from asteroids amended with different types of organic matter. Each bar in (C) and (D) (with the exception of T0 in (C)) is an amalgamation of all respective samples collected over the timecourse. The relative abundance of taxa within asteroid-associated microbiomes varied with sample type. Body wall (i.e. using a biopsy punch; May-June 2018) and epidermis of lesion margin (i.e. scrape on margin; August 2018) assemblages were dominated by orders Spirochaetales and Cytophagales, while assemblages immediately above and on surfaces (collected by swabs), bore a large proportion of orders associated with organic rich environments, e.g., Alteromonadales, Flavobacteriales, Rhizobiales, and Rhodobacterales.

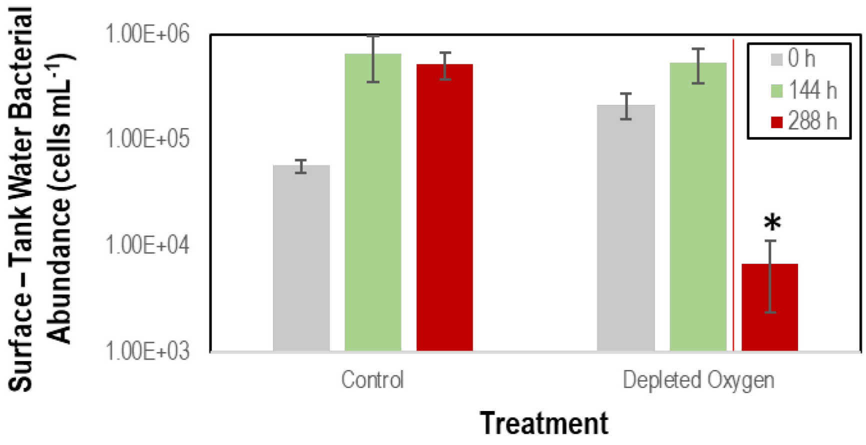

Fig. S8: Bacterial abundance in surface layer above *Asterias forbesi* during depleted O_2 and control treatments (n = 12 each). The abundance was corrected for aquarium water bacterial abundance and measured by SYBR Gold epifluorescence microscopy. The solid vertical red line indicates the average time that suboxic-treated asteroids formed lesions. * denotes $p<0.05$ (Student's *t*-test)

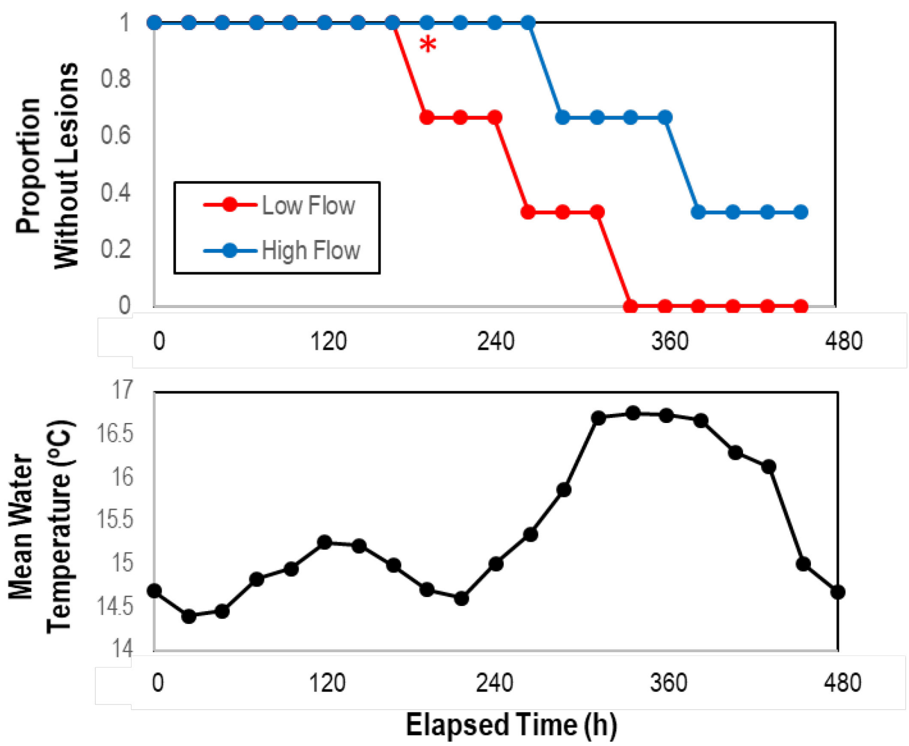

Fig. S9: Proportion asymptomatic (i.e. without lesions) *P. ochraceus* (n = 3) and mean water temperature over time subjected to high or low aquarium water flow rates. * denoted that the time of lesion formation was significantly shorter in the low flow rate incubations than in high-flow incubations (Student's *t*-test).

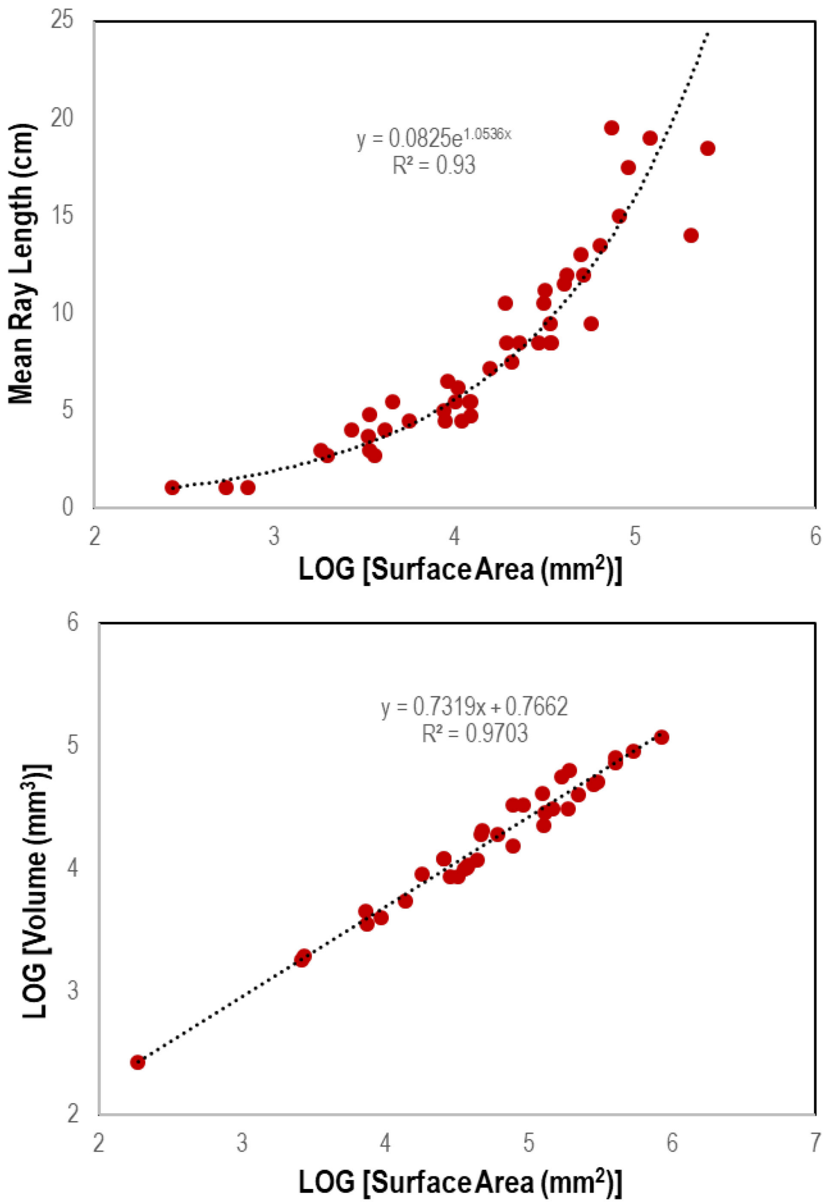

Fig. S10: Relationship between ray length and total surface area, and between volume and surface area of 36 specimens of asteroid.

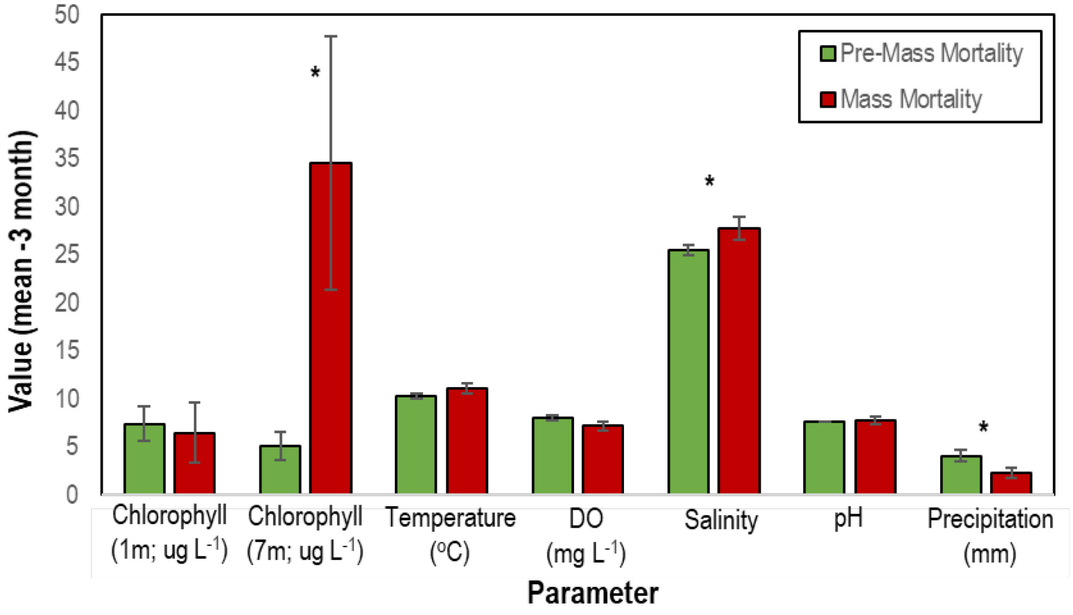

Fig. S11: Comparison of physicochemical, meteorological parameters and chlorophyll a concentration at 1 and 7m depth in the 3 months prior to and including the month of mass mortality with months that did not experience mass mortality. * indicates $p<0.05$.

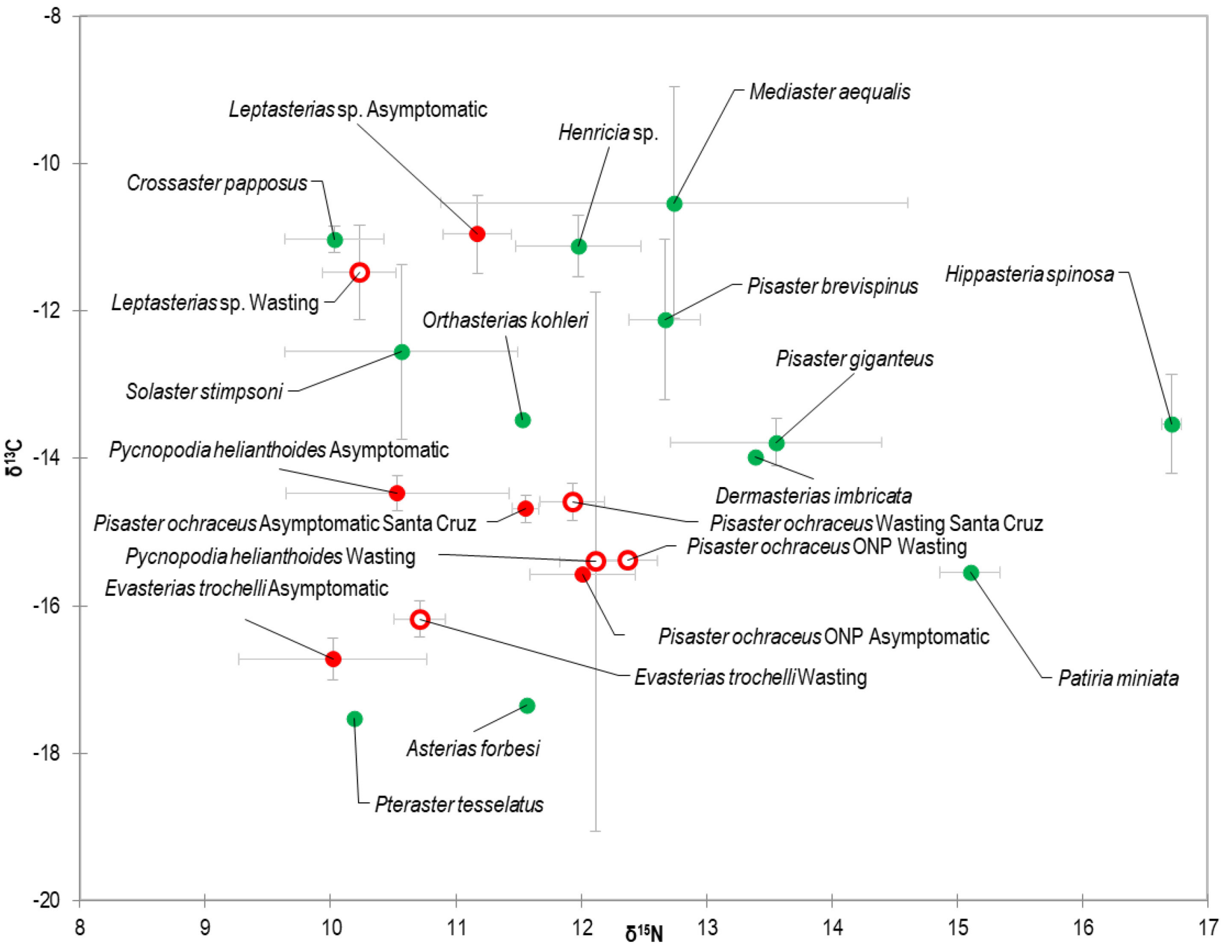

Fig. S12: Stable isotopic ($\delta^{13}C$ and $\delta^{15}N$) composition of asteroids affected by SSW compared to those less affected by wasting. Green data points indicate species for which only grossly normal tissue samples were available, while red symbols indicate samples for which both grossly normal (filled) and wasting-affected (open) tissue samples were analyzed.

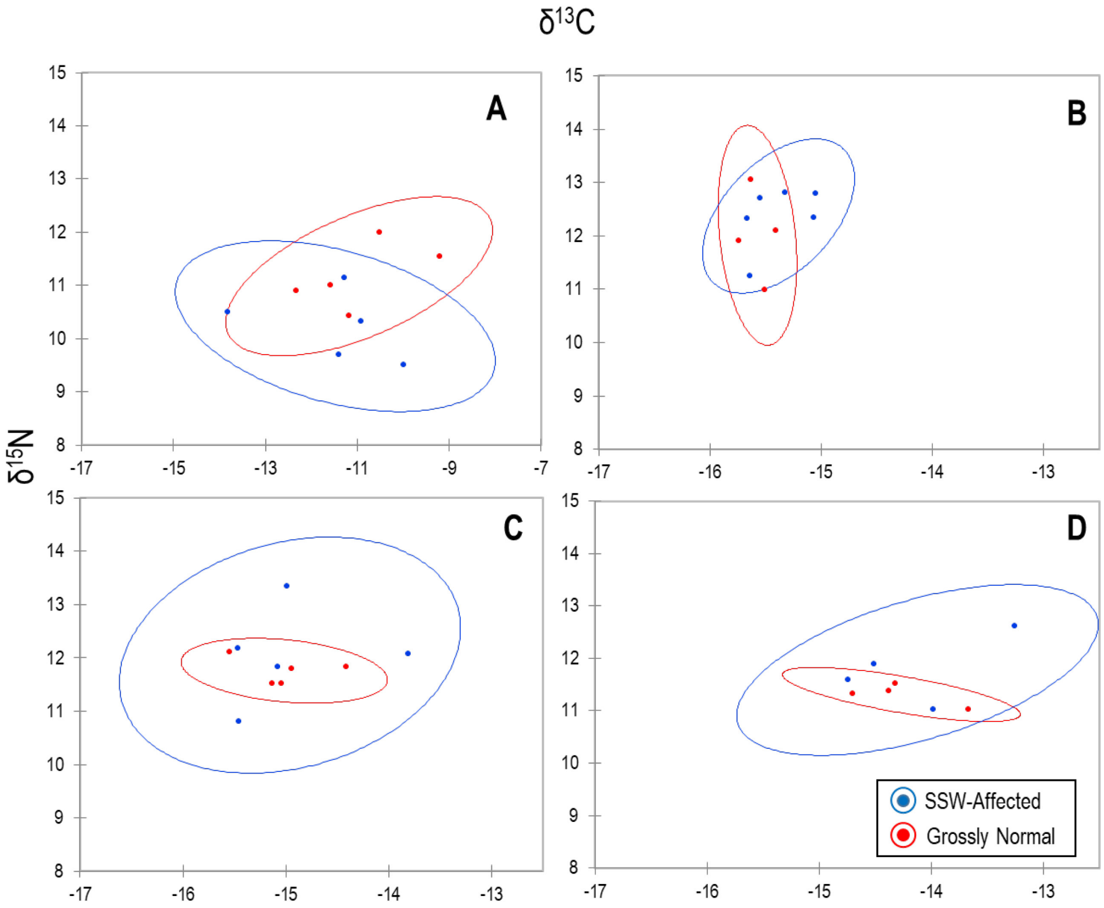

Fig. S13: Comparison of $\delta^{13}C/\delta^{15}N$ between grossly normal and wasting-affected asteroids. Ellipses indicate 95% confidence interval (chi-squared distance). A: *Leptasterias hexactis*, Sitka January 2014; B: *Pisaster ochraceus* Olympic National Park Oct 2013; C: *Pisaster ochraceus* Santa Cruz Oct 2013; D: *Pisaster ochraceus* Olympic National Park Jan 2014

Table S1: Metadata on animal size, mass, aquarium flow rate, and change in mass over time for high/low flow, desiccation and tissue homogenate experiments performed in Santa Cruz, CA, June 2018.

Flow Regime	Treatment	Mass (g)	Ray Length (cm)	Flow Rates (ml s-1)	% Change mass d-1 over experiment	% Change Mass d-1 in First 5 d
High	None	140	8.5	6.697924	-1.681	0.4
High	None	426	13	6.872852	-6.72776	-2.4
High	None	192	10	7.267442	-0.82517	0.4
Low	None	420	13	2.824859	-7.37466	2.8
Low	None	186	9.5	3.532321	-3.18778	0
Low	None	378	13.5	3.078818	-5.84906	-7.2
High	Dessiccation	464	13.5	5.889282	-6.95418	2.8
High	Dessiccation	192	10	8.481764	-3.82749	-0.4
High	Dessiccation	192	11	6.906077	-2.39557	0.4
Low	Dessiccation	492	14.5	1.835536	-8.2459	-19.2
Low	Dessiccation	330	13.5	3.281917	0.8	0.8
Low	Dessiccation	642	14.5	3.313453	-9.77898	-2.4
Low	Homogenized Tissue	438	14	3.443526	-8.06557	6
Low	Homogenized Tissue	554	16.5	3.838772	-4.62295	22.4
Low	Homogenized Tissue	218	10	3.371544	-5.2	-5.2
Low	Proteinase-k Treated Tissue Homogenate	498	13	4.496403	-9.14754	0.4
Low	Proteinase-k Treated Tissue Homogenate	476	11.5	3.846154	-7.50943	3.2
Low	Proteinase-k Treated Tissue Homogenate	180	9.5	3.772161	-4.5876	1.2

Table S2: Model parameters used to study interaction between measured variables and lesion time.

**Model Parameters**	**Parameter Description**	***P. ochraceus* - Initial Wasting**	***P. ochraceus* - Flow Rate**	***P. ochraceus* - OM**	***A. forbesi*-Oxygen**
Lesion Time	Time from initiation of experiment to first appearance of lesion	X	X	X	X
Mass	Mass of animal	X	X	X	X
Ray Length	Average length of ray	X	X		
Aquarium Flow Rate	Flow rate of seawater into incubation container		X		
Overall Mass Loss	Mass lost over the period of the experiment		X	X	X
3d Mass Loss	Mass lost over first 3 days of incubation			X	X
5d Mass Loss	Mass lost over the first 5 days of incubation		X		
Surface Area	Surface area of individual (measured by computed tomography)	X	X	X	X
Surface Area:Volume	Surface area : Volume of individual (measured by computed tomography)	X	X		X
Respiration Rate	Respiration rate of individual measured at experiment initiation			X	X
Measured:Hypothetical Diffusion	Respiration rate as a ratio of calculated hypothetical diffusion rate			X	X
Initial Bacterial Abundance	Bacterial abundance at experiment initiation			X	X
3d Bacterial Abundance	Bacterial abundance change in first 3 d of experiment			X	
Maximum Bacterial Abundance	Maximum bacterial abundance during experiments			X	X

Table S3: Species and metadata for intact whole animal specimens collected for computed tomography.

**Species**	**Date of Collection**	**Location of Collection**	**Latitude (ºN)**	**Longitude (ºW)**	**Ray length of Individual (cm)**
Asterias forbesi	5/29/2019	Bar Harbor, ME	44.427558	68.199568	7.5
Dermasterias imbricata	9/4/2019	Sitka, AK	57.076134	135.41194	3
Dermasterias imbricata	9/4/2019	Sitka, AK	57.076134	135.41194	8.5
Dermasterias imbricata	9/4/2019	Sitka, AK	57.076134	135.41194	5
Dermasterias imbricata	9/4/2019	Sitka, AK	57.098892	135.40156	8.5
Dermasterias imbricata	9/4/2019	Sitka, AK	57.098892	135.40156	6
Dermasterias imbricata	9/4/2019	Sitka, AK	57.098892	135.40156	4.5
Dermasterias imbricata	9/4/2019	Sitka, AK	57.076134	135.41194	5.5
Dermasterias imbricata	9/4/2019	Sitka, AK	57.076134	135.41194	11.25
Dermasterias imbricata	9/4/2019	Sitka, AK	57.076134	135.41194	7.25
Evasterias troschelii	9/4/2019	Sitka, AK	57.076134	135.41194	3.5
Evasterias troschelii	10/1/2019	Friday Harbor, WA	48.595637	122.98137	19
Evasterias troschelii	9/4/2019	Sitka, AK	57.076134	135.41194	11
Evasterias troschelii	9/4/2019	Sitka, AK	57.076134	135.41194	3
Evasterias troschelii	9/4/2019	Sitka, AK	57.076134	135.41194	10.5
Evasterias troschelii	9/4/2019	Sitka, AK	57.076134	135.41194	2
Evasterias troschelii	10/8/2019	Friday Harbor, WA	48.41732	122.66429	19.5
Henricia leviuscula	9/4/2019	Sitka, AK	57.076134	135.41194	4
Henricia leviuscula	9/4/2019	Sitka, AK	57.076134	135.41194	4.5
Henricia leviuscula	9/4/2019	Sitka, AK	57.076134	135.41194	5
Henricia leviuscula	9/4/2019	Sitka, AK	57.076134	135.41194	3.75
Leptasterias sp.	9/21/2019	Santa Cruz, CA	36.626212	121.91517	1
Leptasterias sp.	9/21/2019	Santa Cruz, CA	36.626212	121.91517	1
Leptasterias sp.	9/21/2019	Santa Cruz, CA	36.626212	121.91517	0.5
Orthasterias kohleri	9/4/2019	Sitka, AK	57.076134	135.41194	6
Orthasterias kohleri	9/4/2019	Sitka, AK	57.076134	135.41194	8.5
Orthasterias kohleri	9/4/2019	Sitka, AK	57.076134	135.41194	9.5
Patiria miniata	9/21/2019	Santa Cruz, CA	36.626212	121.91517	4.25
Patiria miniata	9/21/2019	Santa Cruz, CA	36.626212	121.91517	4
Patiria miniata	9/21/2019	Santa Cruz, CA	36.626212	121.91517	4.5
Patiria miniata	9/21/2019	Santa Cruz, CA	36.626212	121.91517	2.75
Pisaster brevispinus	10/8/2019	Friday Harbor, WA	48.41732	122.66429	13.5
Pisaster brevispinus	5/24/2016	Whidbey Island, WA	48.038582	122.40399	5.5
Pisaster giganetus	9/21/2019	Santa Cruz, CA	36.626212	121.91517	17.5
Pisaster giganteus	9/21/2019	Santa Cruz, CA	36.626212	121.91517	15
Pisaster giganteus	9/21/2019	Santa Cruz, CA	36.626212	121.91517	23

Pisaster ochraceus	9/4/2019	Sitka, AK	57.098892	135.40156	10.75
Pisaster ochraceus	9/4/2019	Sitka, AK	57.098892	135.40156	10
Pisaster ochraceus	9/4/2019	Sitka, AK	57.098892	135.40156	8
Pisaster ochraceus	9/4/2019	Sitka, AK	57.098892	135.40156	11
Pisaster ochraceus	9/4/2019	Sitka, AK	57.098892	135.40156	13
Pycnopodia helianthoides	9/4/2019	Sitka, AK	57.076134	135.41194	4.5
Pycnopodia helianthoides	9/4/2019	Sitka, AK	57.076134	135.41194	15
Pycnopodia helianthoides	9/4/2019	Sitka, AK	57.076134	135.41194	8.5
Solaster stimpsoni	10/1/2019	Friday Harbor, WA	48.595637	122.98137	18.5

Table S4: Species and sampling characteristics of individuals used to compare natural abundance of ^{15}N and ^{13}C between wasting-affected and grossly normal tissue states and between species.

**Species**	**Sampling Date**	**Sampling Location**	**Disease State**	**# Specimens**
Pisaster ochraceus	10/23/2013	Starfish Point, Olympic NP	Grossly Normal	5
Pisaster ochraceus	10/23/2013	Starfish Point, Olympic NP	Wasting Affected	5
Pisaster ochraceus	1/3/2014	Starfish Point, Olympic NP	Grossly Normal	5
Pisaster ochraceus	1/3/2014	Starfish Point, Olympic NP	Wasting Affected	6
Pisaster ochraceus	10/8/2013	Davenport, CA	Grossly Normal	5
Pisaster ochraceus	10/8/2013	Davenport, CA	Wasting Affected	5
Pycnopodia helianthoides	10/17/2013	Gibbons, BC, Canada	Grossly Normal	2
Pycnopodia helianthoides	10/16/2013	Gibbons, BC, Canada	Wasting Affected	2
Evasterias trochellii	10/17/2013	Gibbons, BC, Canada	Wasting Affected	3
Evasterias trochellii	10/17/2013	Gibbons, BC, Canada	Grossly Normal	2
Leptasterias hexactis	9/10/2014	Sitka, AK	Grossly Normal	5
Leptasterias hexactis	9/10/2014	Sitka, AK	Wasting Affected	5
Solaster stimpsoni	1/8/2016	Blakley Is, WA	Grossly Normal	2
Patiria miniata	1/23/2014	Monterey, CA	Grossly Normal	2
Crossaster papposus	1/10/2016	Clinton, WA	Grossly Normal	2
Dermasterias imbricata	1/11/2016	Clinton, WA	Grossly Normal	1
Henricia sp.	1/10/2016	Clinton, WA	Grossly Normal	2
Hippasteria spinosa	1/6/2016	West Point, WA	Grossly Normal	2
Mediaster aequalis	1/7/2016	Clinton, WA	Grossly Normal	2
Orthasterias kohleri	1/10/2016	Port Townsend, WA	Grossly Normal	1
Pisaster brevispinus	2/14/2014	Carpinteria, CA	Grossly Normal	2
Pisaster giganteus	3/1/2014	Goleta Pier, CA	Grossly Normal	2
Pteraster tesselatus	1/8/2016	Friday Harbor, WA	Grossly Normal	1
Asterias forbesi	10/3/2013	Bar Harbor, ME	Grossly Normal	2

Table S5: Significance of survival (time to lesion development) in asteroids subjected to treatment under high and low flow regimes. Statistical significance assessed by comparing hazard rate by log rank test.

	**High Flow**	**High Flow - Desiccated**	**Low Flow**	**Low Flow - Desiccated**	**Low Flow - Tissue Homogenate**	**Low Flow - Proteinase-k treated Tissue Homogenate**
**High Flow**	-	ns	ns	0.0389	0.0246	0.0224
**High Flow - Desiccated**		-	ns	0.0389	0.0246	ns
**Low Flow**			-	0.0389	ns	ns
**Low Flow - Desiccated**				-	0.0389	0.0389
**Low Flow - Tissue Homogenate**					-	ns
**Low Flow - Proteinase-k treated Tissue Homogenate**						-

Table S6: Mass-normalized respiration rates, mean ray length and mean animal mass of asteroids, and ratio of measured to maximum hypothetical diffusion rates. Two non-asteroid species are included for comparison. Respiration rates of both *Asterias forbesi* and *Pisaster ochraceus* were higher than for both *Dermasterias imbricata* and *Patiria miniata* ($p<0.0125$; Student's t-test with Bonferroni correction).

**Species**	***n***	**Respiration Rate (μmol kg^{-1} h^{-1})**	**Ray Length (cm)**	**Mass (g)**	**Measured:Maximum Diffusion Rate**
Asterias forbesi	24	1287.2 ± 147.2	7.7 ± 0.5	63.4 ± 6.9	2.8 ± 0.5
Pisaster ochraceus	20	590.3 ± 26.6	11.9 ± 0.4	303.6 ± 28.5	2.7 ± 0.2
Dermasterias imbricata	2	126.1 ± 60.1	7.7 ± 0.1	196.0 ± 10.0	0.7 ± 0.3
Patiria miniata	2	46.5 ± 37.0	7.8 ± 0.1	182.0 ± 20.0	0.6 ± 0.5
